# In Search of the Role of Three-Finger Starfish Proteins

**DOI:** 10.3390/md22110488

**Published:** 2024-10-30

**Authors:** Ekaterina N. Lyukmanova, Maxim L. Bychkov, Andrei M. Chernikov, Ilya D. Kukushkin, Dmitrii S. Kulbatskii, Sergey V. Shabelnikov, Mikhail A. Shulepko, Ran Zhao, Wenxiao Guo, Mikhail P. Kirpichnikov, Zakhar O. Shenkarev, Alexander S. Paramonov

**Affiliations:** 1 Shenzhen MSU-BIT University, No. 1, International University Park Road, Dayun New Town, Longgang District, Shenzhen 518172, China; 2 Shemyakin-Ovchinnikov Institute of Bioorganic Chemistry, Russian Academy of Sciences, Miklukho-Maklaya Str. 16/10, 119997 Moscow, Russia; 3 Moscow Center for Advanced Studies, 123592 Moscow, Russia; 4 Interdisciplinary Scientific and Educational School of Moscow University «Molecular Technologies of the Living Systems and Synthetic Biology», Faculty of Biology, Lomonosov Moscow State University, Leninskie Gory, 119234 Moscow, Russia; 5 Institute of Cytology, Russian Academy of Sciences, Tikhoretsky Prospect 4, 194064 St. Petersburg, Russia

**Keywords:** Lystar5, three-finger protein, LU domain, Ly6/uPAR, Asterias, starfish, adhesion, integrin

## Abstract

Three-finger proteins (TFPs), or Ly6/uPAR proteins, are characterized by the beta-structural LU domain containing three protruding “fingers” and stabilized by four conserved disulfide bonds. TFPs were initially characterized as snake alpha-neurotoxins, but later many studies showed their regulatory roles in different organisms. Despite a known expression of TFPs in vertebrates, they are poorly studied in other taxa. The presence of TFPs in starfish was previously shown, but their targets and functional role still remain unknown. Here, we analyzed expression, target, and possible function of the Lystar5 protein from the *Asterias rubens* starfish using bioinformatics, qPCR, and immunoassay. First, the presence of Lystar5 homologues in all classes of echinoderms was demonstrated. qPCR revealed that mRNA of Lystar5 and LyAr2 are expressed mainly in coelomocytes and coelomic epithelium of *Asterias*, while mRNA of other TFPs, LyAr3, LyAr4, and LyAr5, were also found in a starfish body wall. Using anti-Lystar5 serum from mice immunized by a recombinant Lystar5, we confirmed that this protein is expressed on the surface of coelomocytes and coelomic epithelium cells. According to ELISA, a recombinant analogue of Lystar5 bound to the membrane fraction of coelomocytes and coelomic epithelium but not to the body wall or starfish arm tip. Analysis by LC-MALDI MS/MS suggested integrin α-8-like protein expressed in the coelomocytes and coelomic epithelium as a target of Lystar5. Thus, our insights propose the important role of TFPs in regulation of starfish physiology and show prospects for their further research.

## 1. Introduction

Ly6/uPAR proteins, also referred to as “three-finger proteins” (TFPs), belong to the protein family whose members contain 60–90 amino acid “LU domain(s)” composed of three loops protruding outward from the β-structured core stabilized by the system of conservative disulfide bonds [1,2,3]. 

TFPs were discovered in the wide diversity of species and show a remarkable variety of amino acid sequences that mediate the diversity of their functions. TFPs can be secreted or tethered to a membrane by a GPI-anchor or transmembrane helices [4]. Also, some proteins contain multiple LU domains within one amino acid chain [5,6]. In some cases, LU domains can serve as a functional part of big transmembrane receptors, for example, protein kinases from the transforming growth factor beta (TGFβ) receptor family, including activin (ACVR) and bone-morphogenic protein (BMPR) receptors [4,7,8,9]. Initially, TFPs were discovered as three-finger neurotoxins from snake venoms, which interact with different essential receptors, including nicotinic acetylcholine receptors (nAChRs), and this interaction often results in the receptors’ function inhibition. Their structure [10,11,12], molecular targets [13], and evolution [14,15] are well-studied. Later, TFPs were discovered in mammals, where they play important roles in many vital processes, for example, in the regulation of the plasminogen activation system [5,6,16] and complement system [17] or in the modulation of viral infection [18,19]. However, the most studied functional role of TFPs is the modulation of the cholinergic system. An ability to modulate the neuronal receptors, including nAChRs, is shown for several human TFPs (Lynx1, Lynx2 (Lypd1), Lypd6, Lypd6b, SLURP-1, SLURP-2) [20,21,22,23,24]. This points to human TFPs as potential targets or drug prototypes for treatment of different diseases related to these receptors’ dysfunction [25,26]. 

In contrast to the mammalian and snake TFPs, the TFPs from other taxa are poorly studied. Nevertheless, all of the known non-mammalian TFPs play a crucial role in a variety of physiological processes. For example, the protein Bouncer from zebrafish is the key species-specific female fertilization factor [27]. Expression of few Ly2.1–3 TFPs from *Danio rerio* zebrafish was demonstrated in different tissues with possible involvement in innate immunity and digestive system [28]. The *Lypc* gene, encoding the TFP with unknown function, is expressed in a rhythmic light-dependent pattern in pigment cells of *Danio rerio*. Newt and salamanders have a unique ability to regenerate damaged or amputated limbs that is mediated by another TFP called Prod1 [29,30]. Finally, the TFPs were characterized in some invertebrate. Thus, *Drosophila melanogaster* has a gene encoding the TFP, which organizes epithelial cells to maintain the blood–brain barrier integrity [31]. *Drosophila* sleep is also regulated by the TFP-coding gene *sleepless* [32]. 

Thus, the TFPs have been found in different organisms; however, their taxonomic affiliation is limited mainly to vertebrates and, to a lesser extent, arthropods. TFPs in other types of animals are practically not characterized. Nevertheless, the study of these proteins is important for the understanding of evolutional relationships, physiology and signaling pathways regulation, development of new proteins with useful properties, and search for new modulators for precise drug design. From a therapeutic point of view, studies of the functional role of the TFPs in various organisms with different phylogenetic distances to humans are useful for identifying new targets and modes of interaction of biomolecules. 

The species of the Echinodermata phylum, due to their position on the tree of life [33], are of interest for a search for the TFPs, as, on the one hand, they have a significantly simpler organization, and, on the other hand, they are the evolutionary relatively close to the Chordata phylum, which includes mammals, where the largest number of the TFPs are characterized. The presence of the TFPs in *Asterias rubens* (European starfish) was previously demonstrated by proteomics methods (Lystar1–Lystar4) [34]. We identified additional genes encoding the TFPs in two closely related starfishes, *Asterias rubens* and *Acanthaster planci*. For one of those TFPs (Lystar5, having ~50% sequence similarity with the human Lynx2 protein), expression on a mRNA level in coelomocytes was confirmed [35]. Production of a recombinant analogue of Lystar5 made it possible to study its structure and activity on human neuronal nAChRs. However, the functional role of this TFP in the starfish organism remains unclear.

Here, we studied the possible function of the TFPs and, in particular, Lystar5 in echinoderms. To do this, using sequence searches, 48 TFPs were discovered in the genome of *A. rubens*. The obtained sequences of the LU domains of *A. rubens* were compared with the human LU domains. Homologues for the Lystar5 protein were found in all available transcriptomes of all classes of echinoderms, showing the greatest similarity to the homologues among the Asteroidea (starfishes) class. For the proteins demonstrating the greatest similarity to the human TFPs, the expression of their mRNA in various tissues of *A. rubens* was shown. Then, using qPCR, we revealed the expression of the genes encoding Lystar5 and earlier detected protein LyAr2 mainly in coelomocytes and coelomic epithelium of *A. rubens,* while the expression of the genes encoding another starfish TFP (LyAr3, LyAr4, and LyAr5) was found in the body wall and arm tip. Next, using anti-Lystar5 serum from mice immunized by recombinant Lystar5, we found that Lystar5 is expressed on the surface of coelomocytes and coelomic epithelium cells. Recombinant analogue of Lystar5 bound to membrane fraction of coelomocytes and coelomic epithelium with nanomolar affinity. To identify the possible molecular target of Lystar5, we performed affinity extraction and demonstrated by proteomics that Lystar5 binds integrin α-8-like protein, which regulates cell adhesion. We proposed that Lystar5 is expressed in coelomic epithelium cells to modulate the pro-adhesive activity of integrin α-8-like protein and promote detachment of coelomocytes from the extracellular matrix and their further release into circulation liquid.

## 2. Results

### 2.1. Identification of Proteins Containing the LU Domains in Asterias Rubens Genome

Earlier, we identified several TFPs in the genomes of two starfishes: nine proteins in *Asterias rubens* and six proteins in *Acanthaster planci* [34,35]. For an extended search for the TFPs in the genome of *A. rubens*, we used the sequences of the LU domains of the TFPs previously identified by proteomics [34] and bioinformatics methods [35]. The obtained set was used as a template to search similar sequences using hidden Markov models (HMM) in the set of translated protein sequences from the *A. rubens* genome, available through NCBI. 

As a result, 48 protein sequences corresponding to the starfish proteins containing the LU domains were identified (Table S1). Among these sequences, there were two proteins with GPCR-like architecture (XP_033635539.1 and LyAr2 found earlier, [35]), containing one LU domain at the extracellular *N*-terminal part. Two proteins (XP_033624296.1 and XP_033629184.1) shared the structure with the activin receptor, which is a type I membrane receptor containing an intracellular kinase domain and extracellular LU domain. Three proteins containing two LU domains were also discovered: XP_033629417.1, XP_033638407.1, and XP_033639165.1. The rest of the detected sequences contained one LU domain. 

All proteins were analyzed for the presence of a signal peptide sequence, GPI-anchor attachment sites, and the presence of transmembrane segments (Table S1). Nine proteins did not contain the sites for GPI-anchoring, and seven of them contained predicted TM segments (including two GPCR-like and two activin receptor-like). The rest two (XP_033639165.1 and XP_033633714.1) are likely secreted. Notably, the protein XP_033639165.1 has two LU domains.

### 2.2. Comparison of A. rubens TFPs with Known Human TFPs

Discovery of the TFPs in starfishes indicates that these proteins may play a valuable role in their physiology as they do in other animals. However, to date, no data about the function of the starfish TFPs exist. Comparison of the structures of the proteins from different organisms can help to understand their function and the role in the organism. 

**Figure 1:**
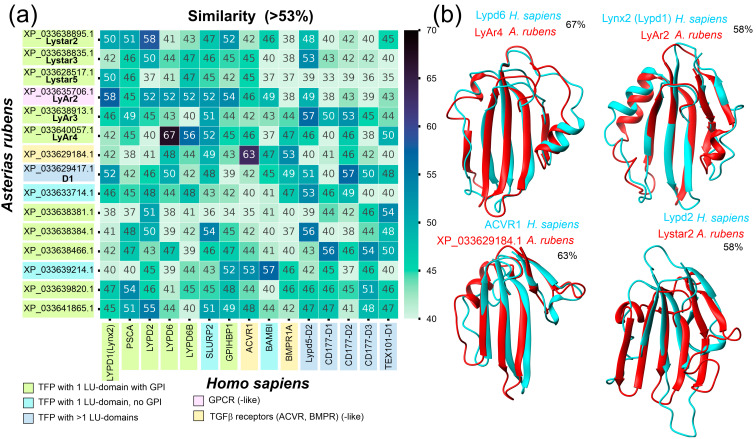
Comparison of human and *A. rubens* TFPs. (**a**) Heatmap of pairwise sequence similarity of the LU domain sequences of the human and *A. rubens* TFPs. Only starfish proteins having high similarity (>53%) with human proteins are shown. (**b**) Superimposition of spatial structures of the LU domains of the human and *A.sterias rubens* TFPs with the highest sequence similarity in ribbon representation. Protein names and sequence similarity (%) are shown on the panels. Experimental NMR structures of Lypd6 and Lynx2 (PDB: 6IB6 and 6ZSS, respectively) are shown. For other cases, sequence-based structures predicted by ESMFold2 [36] are shown.

We compared the sequences of the LU domains of the discovered TFPs from *A. rubens* starfish with the known human TFPs. The choice of human proteins for the comparison was determined by the following considerations: Echinoderms are phylogenetically the closest taxon to chordates, while human proteins are among the best characterized. Moreover, the comparison with the human proteins may reveal the therapeutic potential of the newly discovered TFPs. For this purpose, we selected 46 human proteins containing the LU domains presented in the UNIPROT database. Some of those proteins contain more than one domain (Table S2), and some are membrane receptors. To make the comparison, the sequences of the individual LU domains of *A. rubens* and human proteins were isolated. In total, the sequences of the 51 *A. rubens* and 57 human LU domains were analyzed. To provide an overview of the protein similarity, the pairwise sequence similarity of the LU domains was calculated for starfish and human proteins in the “each to each” manner. Preliminary sequence alignment was carried out for each pair of the LU domains. The similarity calculation results are shown in Figure S1. For some pairs, the noticeably high sequence similarity (more than 50%) was observed. Although, for the most pairs of the starfish and human proteins, the high similarity was not found. Figure 1a shows the reduced similarity matrix, leaving rows and columns that match at least 53% similarity. 

In line with the previous data [35], the highest similarity (67%) was revealed for the LU domain of the human Lypd6 protein and the LyAr4 *A. rubens* protein. Also, high similarity (63%) was found for the LU domains of the human activin receptor type 1 (ACVR1) and starfish protein XP_033629184.1, which is annotated as similar to the vertebrate activin receptor. These two examples show that a number of the TFPs is conserved among evolutionarily distant organisms. Relatively high similarity (58%) was also revealed for the human Lynx2 (Lypd1) protein, representing the single LU domain tethered to a membrane by GPI-anchor, and the LU domain of the starfish LyAr2 protein, which is a GPCR-like membrane receptor. The spatial structures of the LyAr2, LyAr4, and XP_033629184.1 starfish proteins predicted by ESMFold2 [36] were highly similar to those obtained experimentally for the human Lynx2 and Lypd6 proteins and to the predicted structure of the human ACVR1 LU domain, respectively (Figure 1b). However, despite the high sequence similarity between the human Lypd2 and *A. rubens* Lystar2 (XP_033638895.1) proteins, their predicted structures resemble each other quite distantly (Figure 1b). Thus, we observed the high similarity of the previously identified starfish proteins LyAr2 and LyAr4 to the human Lynx2 and Lypd6 proteins, respectively. However, no significant similarity of LyAr5 to any human TFP was found. As previously [35], for LyAr5, we found only 45% amino acid sequence similarity with Ly6D.

**Figure 2:**
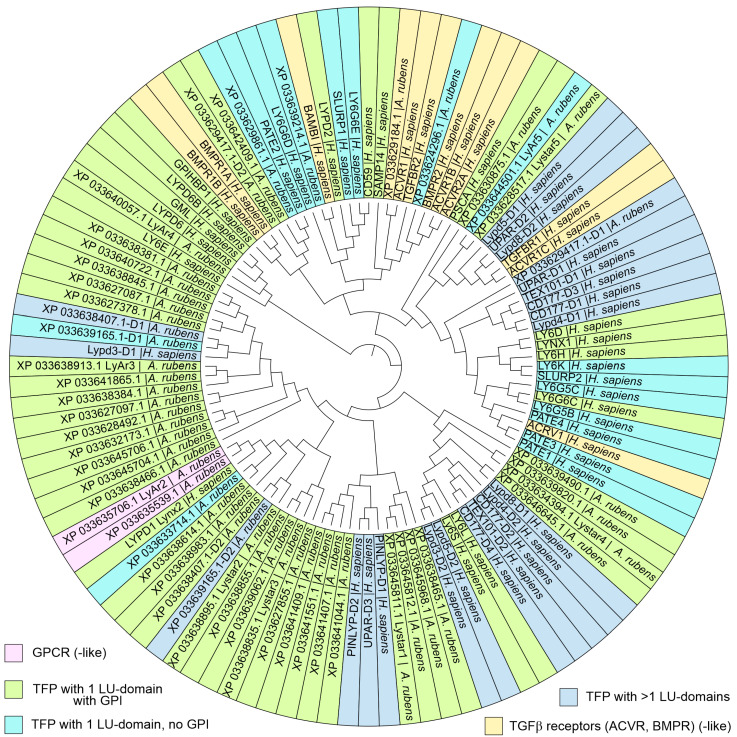
Maximum likehood guide tree of the LU domains of the human and *A. rubens* TFPs. Different types of proteins are signed by colors.

To provide a broad overview of the TFP homology, we built the guide tree (Figure 2) resulting from the multiple sequence alignment (MSA) of the starfish (51 sequences) and human (57 sequences) LU domains. 

### 2.3. Lystar5 Homologues in Echinoderm Species

**Figure 3:**
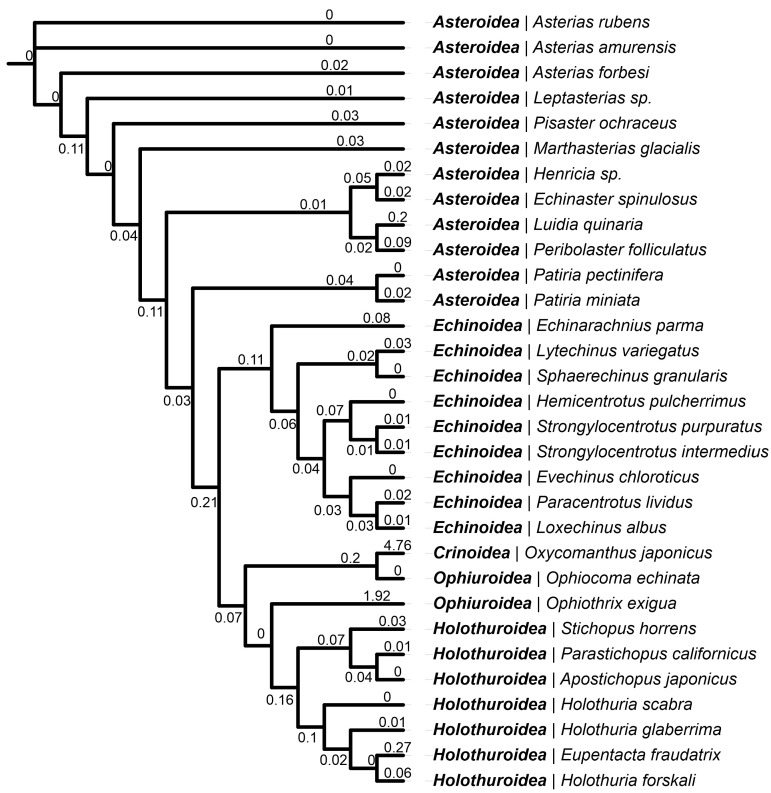
Maximum likehood dendrogram and phylogenetic relationships of *A. rubens* Lystar5 homologues among Echinodermata species. Class and species are shown. Numbers denote similarity distance.

We previously discovered and characterized the three-finger protein Lystar5 from starfish *A. rubens* [35]. To evaluate its importance in the starfish organism, we searched for the homologous of Lystar5 in other echinoderms. The search was carried out using the TBLASTN program among the mRNA sequences in the transcriptome shotgun assemblies (TSA) presented in the NCBI database using the protein sequence of the Lystar5 LU domain as a query. The search was restricted by the Echinodermata phylum. A total of 69 TSA databases were searched in the query, resulting in 110 homologous sequences. Of these, 91 sequences were selected that had 100% sequence coverage by the Lystar5 sequence. From these sequences, the proteins with maximum similarity were selected for each echinoderm species presented in the list. This resulted in the detection of the most similar homologues of Lystar5 in 31 different echinoderm species. According to Table S3, homologues of Lystar5 were found in the members of all classes from the Echinodermata phylum. It should be noted that the sequences with the highest homology (>80%) were found in the Asteroidea (starfishes) class. The similarity for the species from other classes turned out to be less, but still quite high (at least 70%, except for Crinoidea, Table S3). However, for the class Ophiuroidea, the homologues were found only in two species, and the level of homology in one of them *(Ophiothrix exigua*) was less than 50%, which is comparable and less similar to human proteins (see above). Similarly, in the class Crinoidea (sea lilies), the Lystar5 homolog was found only in one species, and the level of sequence homology was also low: 47.3%. This may correspond to the real phylogenetic reasons since Crinoidea is the most distant species from the others (Figure 3), but it may also be a consequence of the low quality of transcriptomic data.

To review the phylogenetic relationships, multiple sequence alignment of 31 protein sequences of the Lystar5 homologues from the echinoderms transcriptomes was aligned, and a dendrogram was built using the maximum likehood method. The resulting dendrogram (Figure 3) generally repeats the phylogeny of the Echinodermata type [37]. In particular, according to the Lystar5 protein sequence, the most distant group was the class Crinoidea. The findings suggest that the Lystar5-like proteins are present in the most echinoderms and possibly play a similar function. 

### 2.4. Expression of the TFPs Genes in Different Tissues of A. rubens on a mRNA Level 

**Figure 4:**
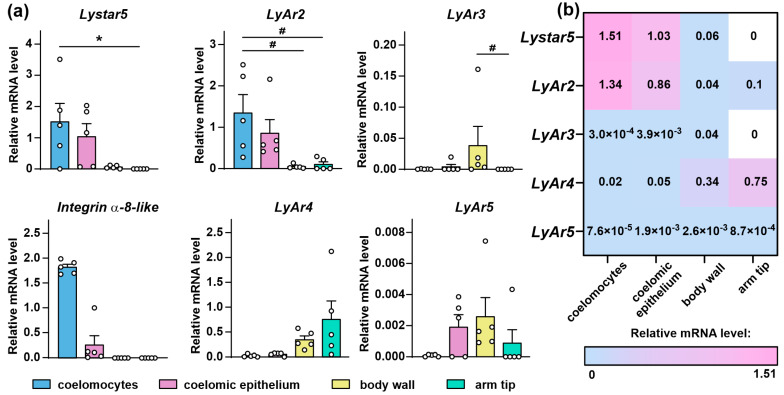
Expression of the TFPs genes and integrin α-8-like gene in different tissues of *A. rubens*: (**a**) Data represented as mRNA level of starfish TFPs normalized to the level of mRNAs encoding 40s ribosomal protein S13 and glyceraldehyde-3-phosphate dehydrogenase ± SEM (n = 5). (* *p* < 0.05) indicates significant difference between the data groups according to one-way ANOVA followed by Tukey’s test. (# *p* < 0.05) indicates a significant difference between the data groups according to the Kruskal–Wallis test followed by Dunn’s test. (**b**) Heatmap representing the mean normalized level of mRNAs encoded different TFPs (Lystar5, LyAr2, LyAr3, LyAr4, and LyAr5).

Earlier, we detected by the BLAST search the TFPs in the *A. rubens* genome [35]. However, the expression on a mRNA level was confirmed only for Lystar5 in the circulatory coelomocytes. Now, to try to understand the TFPs role in Echinodermata, we performed the real-time PCR analysis of expression of the genes encoded the TFPs in different *A. rubens* tissues: circulatory coelomocytes, coelomic epithelium, body wall, and tissue from the arm tip (Figure 4). For the analysis, besides Lystar5, we chose the proteins having the significant sequence similarity (>53%) with the sequences of the human proteins: LyAr2 (XP_033635706), LyAr3 (XP_033638913.1), and LyAr4 (XP_033640057.1) (Figure 1). Additionally, LyAr5 (XP_033644501.1) demonstrating low similarity with the sequences of the human TFPs (<46%, Figure S1) was taken for comparison. 

*Lystar5* and *LyAr2* mRNA were presented mainly in the coelomocytes and coelomic epithelium; however, *LyAr4* mRNA was mainly detected in the body wall and arm tip. The highest level of *LyAr3* mRNA was observed in the body wall, although its mRNA level was more than eight times lower than of *LyAr4*. Trace amounts of *LyAr5* mRNA were found in all tissues studied, while *Lystar5* and *LyAr3* mRNA were not detected at all in the arm tip (Figure 4). 

### 2.5. Lystar5 Is Expressed in Coelomocytes and Coelomic Epithelium on a Protein Level 

**Figure 5:**
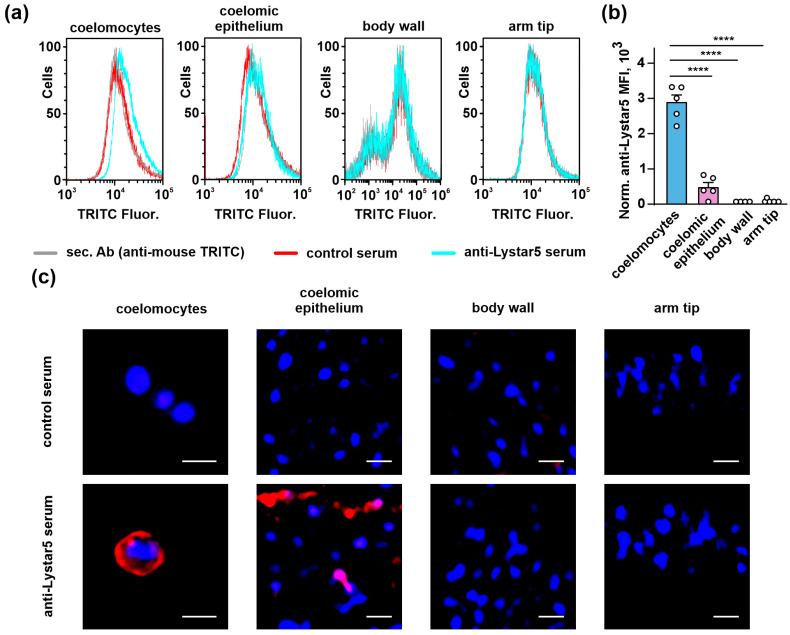
Analysis of Lystar5 expression and localization in different *A. amurensis* tissues: coelomocytes, coelomic epithelium cells, cells of the body wall, and arm tip. (**a**) Representative flow cytometry histograms of cells’ distribution by fluorescence intensity of fluorescent dye (TRITC) conjugated with secondary antibodies. (**b**) Quantification of the Lystar5 expression in different tissues revealed from flow cytometry data. Data presented as normalized MFI ± SEM (n = 4–5). (**** *p* < 0.0001) indicate a significant difference between the data groups according to the one-way ANOVA followed by Tukey’s test. The shift of the median of the cells’ distribution histogram to the right in comparison to control serum indicates the specific binding of anti-Lystar5 serum to analyzed cells. (**c**) Lystar5 localization in the cells of different tissues assayed by confocal microscopy (n = 5). Scale bar = 10 µm; nuclei were stained by Hoechst 33342.

From here, we focused our present study on Lystar5, as previously we received the structural and functional data for it, leaving other starfish proteins for further studies. To confirm the tissue-specific Lystar5 expression on a protein level, we obtained anti-Lystar5 serum from mice immunized with the recombinant analogue of the protein. The serum obtained from mice immunized with bovine serum albumin (BSA) served as a negative control (control serum). We used the fixed cells/tissues of *Asterias amurensis* starfish, which is very close to *A. rubens* [38] and expresses the same Lystar5 sequence (Table S3, Figure S3). In first, we confirmed by real-time PCR the similar mRNA expression profile for Lystar5 in *A. amurensis* as in *A. rubens* (Figure S4). Then, using anti-Lystar5 serum, flow cytometry, and confocal microscopy, we showed that, in accordance with the real-time PCR data (Figure 4), Lystar5 is expressed on the surface of coelomocytes and coelomic epithelium cells while absent in the cells of the body wall and arm tip (Figure 5).

### 2.6. Lystar5 Targets the Membrane Protein in Coelomocytes and Coelomic Epithelium

**Figure 6:**
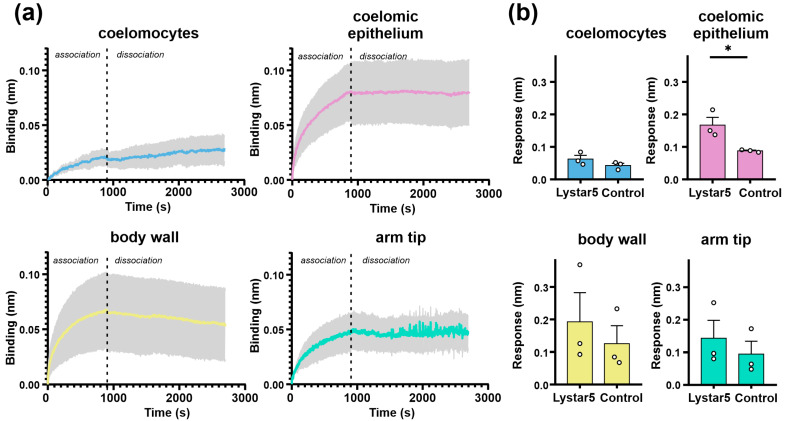
Interaction of immobilized recombinant Lystar5 with lysates of tissues of *A. amurensis* by BLI. (**a**) Traces represent average of three double-referenced (after subtraction of non-specific binding) BLI sensograms for each tissue ± SEM (n = 3). (**b**) Bar graphs represent the mean values for maximal response (at 870 seconds) for BLI biosensors with immobilized Lystar5 and without Lystar5 (control) ± SEM (n = 3). * *p* < 0.05 indicates a significant difference between the responses measured by using the Lystar5-coupled and control biosensors according to the unpaired parametric *t*-test.

After confirmation of the Lystar5 expression on a protein level on the surface of starfish coelomocytes, we suggested that its target is also expressed in the membrane of coelomic cells. To prove it, we immobilized recombinant Lystar5 on the biosensor and studied the interaction of Lystar5 with detergent-solubilized total lysates of different tissues of *A. amurensis* by the bio-layer interferometry (BLI) assay. Empty biosensor was used as a negative control. Some binding was observed not only with the Lystar5-coupled sensor but with the unloaded (control) sensor, too (Figure S6). Absolute values of the binding responses did not exceed 0.4 nm, but in all cases, the binding to the sensor coupled with Lystar5 was stronger than to the control sensor (Figure 6a). A statistically significant difference from the control was observed only for coelomic epithelium (Figure 6b).

**Figure 7:**
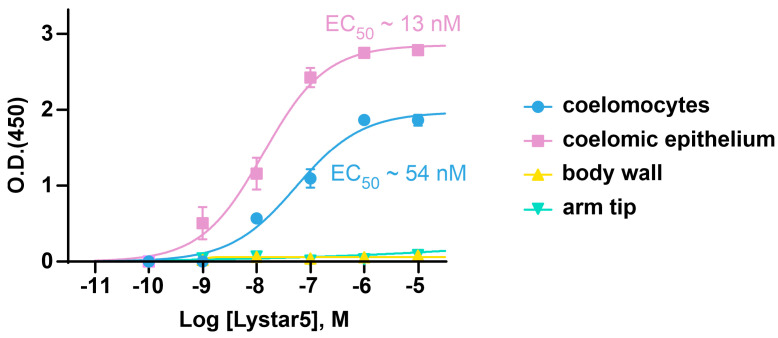
ELISA analysis of the Lystar5 interaction with immobilized membrane fraction of different tissues of *A. rubens*. Parameters describing the curve fit for coelomocytes: A_1_ = 1.97 ± 0.09, EC_50_ = 54.5 ± 15.4 nM, nH = 0.71 ± 0.1; for coelomic epithelium: A_1_ = 2.85 ± 0.12, EC_50_ = 13.4 ± 6.6 nM, nH = 0.75 ± 0.10. Curves were not fitted for the body wall and arm tip. Data present as the background-subtracted data, O.D. ± SEM (n=5).

As we obtained unconvincing BLI data, probably due to the high non-specificity of the binding of tissue lysates to the biosensor or/and the influence of immobilization of Lystar5 on the biosensor on the protein structure and function, we used ELISA to study the interaction of recombinant Lystar5 with its target in the membrane fraction of different *A. rubens* tissues. In the ELISA study, we used the reversed configuration: the membrane fraction of different tissues was immobilized to the plate, while Lystar5 was in solution in different concentrations. ELISA showed that Lystar5 bound to the membrane fraction of coelomocytes and coelomic epithelium with nanomolar affinity (EC_50_~54 nM and ~13 nM, respectively, Figure 7). At the same time, no Lystar5 binding was detected upon its incubation with the membrane fraction of the body wall or arm tip (Figure 7), pointing to the specificity of the data obtained for coelomic cells. 

### 2.7. Integrin α-8-like Protein Is Suggested Lystar5 Target in Coelomocytes and Coelomic Epithelium

To identify possible Lystar5 molecular partners, we conjugated Lystar5 to HNS-activated Sepharose and performed the pull-down assay by extraction of the Lystar5 targets from the membrane fraction of coelomocytes. Empty blocked resin was used as a negative control. Proteins extracted by resins were analyzed by LC-MALDI MS/MS. We identified several proteins extracted by Lystar5-conjugated resin but not by empty resin, although only one of these proteins has a membrane localization: the integrin α-8-like protein (XP_033639394.1, Table S5). 

We analyzed its mRNA expression in the different *A. rubens* tissues and found its expression in the coelomocytes and coelomic epithelium, but not in the samples of the body wall or arm tip (Figure 4a). The fact that integrin α-8-like protein is expressed in the same tissues as Lystar5 indicates that Lystar5 indeed can target it and regulate its function. 

## 3. Discussion

TFPs are present in many organisms and perform important functions. The presence of these proteins was already shown in many taxonomic groups. The most studied TFPs were found in vertebrates, although their presence in more distant taxa, for example, in arthropods or trematodes, was also described [31,32,39]. This means that proteins with the three-finger fold formed quite early in the evolution and may be present in all Metazoa, although it is not directly shown yet. 

The presence of the TFPs in the starfish genomes was shown previously. On a protein level, four TFPs were revealed in the coelomic fluid of the starfish *Asterias rubens* by proteomic analysis [34]. Also, using bioinformatics methods, the presence of the genes encoding the TFPs in the genomes of starfish *Asterias rubens* and *Acanthaster planci* was shown using the BLAST search [35]. In that case, a set of the LU domain sequences of the currently known TFPs was used as a database for BLAST search of the starfish TFPs using a strong detection threshold (E-value 10^−5^). That approach allowed to identify a limited number of starfish TFPs (five for *A. rubens* and six for *A. planci*), but with the highest correspondence to the known TFPs within the used database. For one of those identified proteins (Lystar5), a recombinant production system was developed, and the structural and functional properties of the starfish protein were studied [35]. Here, we performed the broader search, using the LU domain sequences of the TFPs from *A. rubens* as a reference set. Use of the search algorithms based on hidden Markov models allowed us to look more broadly at the TFPs presented in *A. rubens*, and we found 48 proteins containing the LU domains (Table S1). Most of the found sequences of the TFPs were small proteins (mainly up to 150 residues) containing one LU domain, the signal peptide, and a site for the GPI-anchor. Thus, it can be assumed that the most TFPs (if they are expressed on a protein level) are anchored in the cell membrane. A similar situation is observed for the majority of the mammalian TFPs [2,3]. Although there are examples of the mammalian TFPs that can be presented both in membrane-associated and secreted forms (Lynx1 [40], PSCA [41,42,43]). However, such proteins cannot be predicted based on the available data for *A. rubens* starfish. Among the identified starfish TFPs, some proteins already had a putative description obtained through the automated annotation based on similarity with known proteins [44]. In particular, a number of proteins is indicated to belong to the TFP family (Table S1). Several proteins were annotated as the membrane receptors containing the LU domains, such as activin receptor-like and GPCR-like (Table S1). However, the annotation is not provided for the significant part of the sequences.

To predict the functional role of the identified TFPs, we compared the sequences of the LU domains found in *A. rubens* with the domains of the human TFPs by direct calculation of pairwise similarity (Figure 1 and Figure S1) and built a guide tree from MSA (Figure 2). The overview map (Figure S1) showed that in the most cases the similarity does not exceed 50%. However, for several pairs we saw the significantly higher similarity values (55–67%, Figure 1a). For four pairs of the proteins demonstrating the highest similarity, we compared their spatial structures using the known experimental structures (if applicable) or sequence-based predicted ones (Figure 1b). The highest sequence similarity (67%) was observed for the LyAr4-Lypd6 pair, as it was revealed previously [35]. Here, we came to the conclusion that this similarity is the maximum possible compared with other pairs. Lypd6 is the regulator of the Wnt-signaling and cholinergic systems and is a valuable participant in the embryonic development of vertebrates [45] and the mammalian CNS function [46]. The high sequence similarity of LyAr4 from *A. rubens* with human Lypd6 suggests a high conservation of the function of the proteins of this type among different types of animals, apparently indicating an early emergence during phylogenesis. The second characteristic pair with the high similarity of the LU domains (63%) was the human activin receptor (ACVR1) and its homologous starfish protein (XP_033629184.1). Activin receptors are part of the transforming growth factor beta (TGFβ) signaling pathway, which is involved in many cellular processes across all Metazoa [47]. Another pair with the high sequence similarity of the LU domains (58%) was the starfish LyAr2 protein and the human Lynx2 protein. Notably, LyAr2 is a GPCR-like membrane protein having the LU domain on the extracellular *N*-terminal part of the molecule. At the same time, Lynx2 is a small protein consisting of one GPI-anchored LU domain, which is expressed in the mammalian brain, modulates nAChR function, and is associated with anxiety behavior [24]. Since the homologous human GPCR does not have a pronounced LU domain [48,49], it can be assumed that LyAr2 and homologous starfish proteins such as LyAp2 from *A. planci* [35] perform the same function in echinoderms as Lynx2 does in mammals. 

Earlier, we proposed that Lystar5, which demonstrates the greatest similarity to Lynx2 among the human TFPs, is its functional homologue. However, the similarity value (50%) in comparison with the similarities obtained here for other protein pairs (Figure 1 and Figure S1) is not remarkable. Although, similar to Lynx2, Lystar5 modulates the function of human α4β2-nAChR [24,35], it is highly likely that the mechanisms of action of Lystar5 and Lynx2 are different. Indeed, here we did not reveal nAChR-like proteins among the proteins extracted by Lystar5 from the *A. rubens* coelomocytes (Table S5). The broader bioinformatic analysis performed here proposes that nAChR-like proteins in *A. rubens* may be modulated by other TFPs, for example, LyAr2 and LyAr4. 

In an attempt to predict a value of Lystar5 function in the starfish organism, we analyzed the sequences of Lystar5 homologues found in the transcriptomes of various echinoderms. The presence of homologous proteins in representatives of all five echinoderm classes (Figure 3) suggests that the Lystar5 homologues are subject to selection. Moreover, the significant similarity value (70–100%) indicates that the function of this protein in various echinoderms is the same.

Previously, we showed the presence of mRNA encoding Lystar5 in the coelomocytes of *A. rubens* [35]. Here, we extended these data and revealed that Lystar5 is expressed in the coelomocytes and coelomic epithelium of *A. rubens*, while its mRNA is barely absent in the starfish body wall and arm tip (Figure 4). Coelomic epithelial cells play many essential functions in the starfish body. In particular, they participate in immunity-related processes as well as in regeneration processes, which are notable for representatives of echinoderms [50]. In coelomic epithelial cells, the processes of protein synthesis and processing, membrane traffic, and secretion of molecules, including growth and regeneration factors, are noticeably intensified compared with the coelomocytes, which act as immune cells [51]. In addition, the coelomic epithelium serves as a source of the coelomocytes [51,52]. Thus, Lystar5 mRNA expression in the coelomocytes and coelomic epithelium may indicate its relevance for starfish immune responses, tissue regeneration, and other essential processes. 

Here, besides *Lystar5*, we studied the mRNA expression profile for several other TFPs of *A. rubens*: *LyAr2*–*LyAr5*. Interestingly, the expression patterns of *Lystar5* and *LyAr2* were different from those of *LyAr3*, *LyAr4*, and *LyAr5* (Figure 4). *Lystar5* and *LyAr2* were found mainly in the coelomocytes and coelomic epithelium, while *Lyar4* was highly presented in the body wall and arm tip. *LyAr3* and *LyAr5* expression was not very high in comparison with *Lystar5*, *LyAr2*, and *LyAr4* and was uniformly distributed among the studied tissues (Figure 4). Different expression patterns may indicate various physiological roles of the TFPs in *A. rubens*. 

To confirm the Lystar5 expression on a protein level, we produced anti-Lystar5 serum and analyzed its expression in different tissues of *A. amurensis*, a starfish closely related to *A. rubens*, and also expressing Lystar5 (Figure S4). Analysis by flow cytometry and confocal microscopy confirmed the expression of Lystar5 on the membrane of the coelomocytes and coelomic epithelium cells (Figure 5). Previously, we predicted the site for GPI-attachment on the *C*-terminus of Lystar5 [35], and here we showed that indeed this protein is localized on the membrane surface of the coelomocytes. Interestingly, the Lystar5 expression was ~6 times higher in coelomocytes than in coelomic epithelium (Figure 5). It seems that Lystar5 may have some functions in the coelomic epithelium, which serves as a source of the coelomocytes; however, the Lystar5 function is much more pronounced in the coelomocytes. 

The specific nanomolar affinity of recombinant Lystar5 to the membrane fraction of the coelomocytes and coelomic epithelium (Figure 7) indicates that Lystar5 binds with and maybe modulates the function of some membrane protein in these cells. To identify this target, we performed affinity extraction of possible targets from the membrane fraction of the coelomocytes using Lystar5 as a bait. Among extracted proteins, there was a plethora of soluble proteins and only one membrane protein, integrin α-8-like protein (Table S5). Real-time PCR confirmed tissue-specific expression of mRNA encoding this protein. Similar to *Lystar5*, the *integrin α-8-like* gene was found mainly in the coelomocytes and coelomic epithelium (Figure 4a). Thus, it can be assumed that Lystar5, being a GPI-anchored protein, somehow interacts with an integrin-like membrane protein in starfish. This is consistent with the fact that many studied mammalian GPI-anchored TFPs act on membrane receptors [53,54]. However, our data do not exclude other proteins as possible candidates for the Lystar5 target and leave the field for further research. In humans, integrin α8 is expressed on the surface of mesenchymal cells and binds to the RGD- motif of the extracellular matrix, thus mediating the cell adhesion [55]. Probably, in the case when the integrin α-8-like protein is the Lystar5 target in *A. rubens,* it can also mediate the adhesion of the coelomic epithelial cells and coelomocytes to the matrix, while Lystar5 may limit integrin α-8-like pro-adhesive activity, promoting a detachment of the coelomocytes from the coelomic epithelium and their further release to the pool of circulating cells. This assumption seems to be quite speculative, and additional investigations of the Lystar5 function and physiological role in *Asterias* organisms are required. 

Notably, many TFPs in humans serve as auxiliary regulators of membrane receptors, e.g., nAChRs [54]. Our data indicate that the three-finger fold was designed by evolution for the tight control of a plethora of membrane receptors, and TFP function could not be restricted only to nAChR modulation in mammals. The “general paradigm” of the TFP function—modulation of activity of a membrane receptor by GPI-tethered TFP—is preserved upon evolution. Further analysis of structure and pharmacology of the *Asterias* TFPs can give new information about the physiology of starfishes and could provide new modulators of human receptors.

## 4. Materials and Methods

### 4.1. Bioinformatics Analysis

Search of new TFPs in *Asterias rubens* was performed among the translated protein sequences obtained from the *A. rubens* reference genome (NCBI RefSeq assembly GCF_902459465.1). The sequences of the LU domains of the TFPs discovered in *A. rubens* by proteomics [34] (Lystar1 (XP_033645811.1), Lystar2 (XP_033638895.1), Lystar3 (XP_033638835.1), Lystar4 (XP_033634394.1), and detected in the genome by the BLAST search [35] (LyAr1 (Lystar5)–LyAr5, Table S1, ##1–9) were aligned using the MAFFT software [56]. Obtained alignment was used to build the model by the *hmmbiuld* tool from HH-suite3 package [57]. The search was performed by the *hmmsearch* tool. LU domains were extracted from the obtained sequences using resulting software output together with manual inspection (see supplementary FASTA file). Presence of the signal peptide and GPI anchor were predicted by the analysis of the full protein sequences by SignalP 6.0 [58] and PredGPI [59] services. Transmembrane regions were identified by Phobius [60].

LU domains of the human proteins were extracted from the protein sequences from the UNIPROT database [61] (Table S2, June 2024) using the domain annotation information from UNIPROT together with manual inspection, including the use of 3D structure AlphaFold [62] or ESMFold2 [36] prediction (see supplementary FASTA file). 

Pairwise sequence similarity between the starfish and human LU domains was calculated after global sequence alignment of corresponding pair by built-in function from Biotite [63] package using the modified BLOSUM62 matrix with weight of Cys-Cys of 99. The amino acid residues were divided into groups: hydrophobic (A,F,H,I,L,M,P,V,W), cysteines (C), polar (G,N,Q,S,T,Y), positively (K,R), and negatively (D,E) charged and gaps. The similarity was calculated as the percentage of coincidences of the residues belonging to the same group in each position when comparing gapped aligned sequences. Pairwise similarities matrix was visualized as a heatmap (Figure S1) using Seaborn library [64]. For visualization of the highest similarities, the rows and columns with the maximal similarity value greater than 53% were selected. To this submatrix, the row with the values for Lystar5 was added (Figure 1a). 

For guide tree building, the joined two sets of the LU domains (human and *A. rubens*) were subjected to multiple sequence alignment using MAFFT [56], and guide tree was calculated using IQ-TREE2 software [65]. For tree visualization, the iTool service was used [66].

For search of Lystar5 homologues in the Echinodermata species, the sequence of the LU domain of Lystar5 was submitted to the NCBI BLAST server (https://blast.ncbi.nlm.nih.gov) with setup for search by TBLASTN utility among transcriptome shotgun assemblies (TSA) presented in the NCBI database with restriction by Echinodermata phylum. Translated protein sequences were aligned by MSA using MAFFT [56]. The tree was calculated by IQ-TREE2 [65] and visualized by iTOL [66].

All manipulations were performed by in-house Python scripts using Biopython [67] and Biotite [63] bioinformatic libraries.

### 4.2. Animals and Tissue Collection 

To investigate Lystar5 expression and its target, we used tissue samples of *A. rubens* and *Asterias amurensis*, two closely related starfishes [38].

Adult five individuals of *A. rubens* starfish were collected at the Biological Station of the Zoological Institute, Russian Academy of Sciences, on Cape Kartesh (Kandalaksha Bay, White Sea) in September 2022. Adult *A. amurensis* five individuals were obtained from Shenyu Marine Technology (China). Coelomic epithelium was obtained by cutting out epithelium from aboral side of a starfish, as in [52]. Remains of the starfish arms were used as the body wall. The tissue from the arm tip was obtained by making ~5 mm cut in the area of the eye spot. All tissues were frozen in liquid nitrogen. Circulatory coelomocytes were collected by cutting off the arm tip and collecting the coelomic fluid into a tube with anticoagulant solution [68]. The cells for PCR, ELISA, and affinity purification were pelleted by centrifugation at 120× *g* for 10 min in a bucket rotor and snap-frozen in liquid nitrogen. The coelomocytes and tissue samples for flow cytometry and confocal microscopy were fixed in 4% paraformaldehyde.

### 4.3. Real-Time PCR Analysis of Expression of TFP Genes in Different Tissues of A. rubens

To analyze the expression of mRNA encoded the Lystar5, LyAr1, LyAr2, LyAr3, LyAr4 proteins and integrin α-8-like protein (mRNA accession IDs XM_033772626.1, XM_033779815.1, XM_033783022.1, XM_033784166.1, XM_033788610.1, XM_033788610.1), real-time PCR was used. The total RNA from four types of tissues (coelomic epithelium, circulatory coelomocytes, body wall, and arm tip) was isolated using ExtractRna kit (Evrogen, Moscow, Russia). In total, five samples from different starfish individuals were prepared for each type of tissue. The cDNA was synthesized using Mint cDNA synthesis kit (Evrogen). Real-time PCR was performed on LightCycler 96 amplifier (Roche, Basel, Switzerland) using SYBR Green HS mix (Evrogen, Russia) and specific primers (Table S4). The mRNA level was normalized to the expression of housekeeping genes 40s ribosomal protein S13 and glyceraldehyde-3-phosphate dehydrogenase, mRNA accession IDs XM_033789754.1 and XM_033786142.1, respectively. The stability of the both housekeeping genes expression was confirmed by equal Ct values in all analyzed groups.

### 4.4. Recombinant Lystar5 Expression and Purification

Recombinant analogue of Lystar5 was produced as previously described [35]. The protein was characterized by MALDI-MS and HPLC. Proper spatial structure was confirmed by 1D ^1^H-NMR. 

### 4.5. Mice Immunization

To analyze expression, localization, and Lystar5 interaction with possible molecular targets, we obtained anti-Lystar5 serum from mice immunized by recombinant Lystar5. All animal care and experimental procedures were performed in accordance with the guidelines set forth by the European Communities Council Directive of 24 November 1986 (86/609/EEC) and were approved by the Ethical Committee of IBCH RAS for the control of the maintenance and use of animals (protocol 380/2024).

Two 10-week BALB/c mice were intraperitoneally immunized by 20 µg of recombinant Lystar5 dissolved in 100 µL of complete Freund’s adjuvant (FCA, BD Biosciences, NJ, USA). After 7 days, mice were additionally immunized by 20 µg of recombinant Lystar5. For control, two mice were immunized with BSA (PanEco, Moscow, Russia) by the same protocol. After additional 7 days, mice were euthanized in CO_2_ chamber (Acrylmedic, Romashkovo, Russia), decapitated, and serum was obtained from blood clotted in RT for 30 min. Resulting serum was designated as anti-Lystar5 serum (Lystar5-immunized mice) and control serum (BSA-immunized mice). We used the following strategy for all used immunoassays: (1) secondary control: only secondary anti-mouse conjugated antibodies; those mean fluorescent intensity (MFI) was subtracted from all experimental data; (2) biological control: the serum from mice immunized by BSA+FCA, which was compared with the experimental group.

### 4.6. Flow Cytometry

To analyze the endogenous Lystar5 expression, the fixed coelomocytes or tissues of coelomic epithelium, body wall, or arm tip disintegrated by Accutase (Life Technologies, Waltham, CA, USA) were used. The cells or disintegrated tissue samples were blocked by 1% horse serum in PBS + 0.1% Tween 20 for 1 h, incubated with anti-Lystar5 or control serum (see above) for 4 h, washed 3 times, and incubated with goat TRITC conjugated anti-mouse antibody (Jackson Immunoresearch, 115-025-003 1:1000, West Grove, CA, USA) for 1 h, washed 3 times, and analyzed by Attune NxT flow cytometer (Life Technologies). Data were analyzed using Attune NxT Software 2.3 (Life Technologies). In total, five samples from different starfish individuals for each type of tissue were analyzed. The MFI of cells stained with control anti-serum and secondary Abs were subtracted from MFI of cells stained with anti-Lystar5 serum. The gating strategy is in Figure S5.

### 4.7. Confocal Microscopy

Fixed coelomocytes or tissues of coelomic epithelium, body wall, or arm tip from five starfish individuals were used for confocal microscopy. First, heat epitope retrieval was performed in Declere buffer (ESBE Scientific, Markham, ON, Canada) for 20 min at 90 °C. After that samples were blocked in 1% horse serum in PBS + 0.1% Tween 20 for 8 h and incubated with anti-Lystar5 or control serum (see above) for 72 h, washed 3 times, incubated with goat TRITC conjugated anti-mouse antibody (Jackson Immunoresearch, 115-025-003, 1:1000, West Grove, CA, USA), washed 3 times, then mounted in mowiol-DABCO and observed under 25× (0.8) objective by Carl Zeiss LSM710 inverted confocal microscope (Jena, Germany). Nuclei were stained by Hoechst 33342.

### 4.8. ELISA

To investigate the interaction of Lystar5 with its target, we used ELISA. Membrane fraction of four different *A. rubens* tissues from four different starfish individuals was isolated as in [69]. After that, membrane fraction was dissolved in sodium bicarbonate buffer (pH = 9.3) and immobilized on the 96-well ELISA plates (9018, Corning, Corning, MS, USA) for 3 h (RT) at concentration of 2 µg of total protein per well. Then, plates were blocked by 5% BSA in PBS for 2 h (RT), washed (ELISA wash buffer, Mybiosource, San Diego, CA, USA), incubated with different concentrations of Lystar5 (10^−5^–10^−10^ M) for 2 h, washed, incubated with anti-Lystar5 serum (1:100), for 1 h, washed, incubated with donkey anti-mouse HRP-conjugated antibody (Jackson Immunoresearch, 715-035-150, 1:1000) for 1 h, washed, and incubated with TMB-One ELISA Substrate (Mybiosource). The reaction was stopped by 200 mM HCl, and O.D. was measured using AMR-100 microplate reader (Allsheng, Hangzhou, China) at 450 nm. The O.D. from the wells incubated with PBS was subtracted from O.D. of the wells incubated with recombinant Lystar5. The O.D. curves were fitted using the Hill equation of GraphPad Prism 8.0 software (GraphPad Software, San Diego, CA, USA).

### 4.9. Affinity Extraction

To identify the Lystar5 target in the coelomocytes, the recombinant Lystar5 was coupled to NHS-activated Sepharose 4 Fast Flow (Cat #17-0906-01, GE Healthcare, Chicago, IL, USA) according to the manufacturer’s instructions. The resin was blocked by 500 mM ethanolamine without any protein coupled and was used as a negative control (empty resin). The membrane fraction of the coelomocytes was obtained as in [69] and incubated with the resin for 16 h at 4 °C. After that, non-specifically bound proteins were sequentially washed out from the resin with five volumes of phosphate-buffered saline (PBS) + 1 M NaCl + 0.5% Triton X-100 and five volumes of PBS + 0.5% Triton X-100. Then, the resin was incubated with 8M urea, 5 mM DTT, 50 mM TRIS, and pH 8.0 for elution of proteins bound to Lystar5. Elution was performed twice with buffer change. Four samples of membrane fractions for both types of resin (control and Lystar5-conjugated) were subjected to extraction for subsequent proteomic analysis.

### 4.10. Protein Digestion and Peptide Fractionation

Proteins eluted from resin were digested with trypsin. All digests were desalted and concentrated with Strata-X 30 mg solid-phase extraction tubes (Phenomenex, CA, USA). Eluted digests were supplemented with 10 μL of 50 mg/mL D-glucose to aid subsequent rehydration and dried with a rotor vacuum evaporator. Then, digests were resuspended in 25 mL of 1% (*v*/*v*) formic acid in water and filtered through 0.2 μm PVDF filter. Peptides were separated with a Chromolith CapRod RP-18e HR reversed-phase column (0.1 mm × 150 mm, Merck, Darmstadt, Germany) on a nano LC system (Eksigent NanoLC Ultra 2D+ system, SCIEX, Darmstadt, Germany). A total peptide amount of 1000 ng was loaded and separated using a linear gradient of 6.5–43% B over 44 min at a flow rate of 400 nL·min^−1^. The mobile phases used were A: water with 0.2% (*v*/*v*) TFA and B: 80% (*v*/*v*) acetonitrile in water. The column was operated at a room temperature of 22–24 °C. The effluent from the column was mixed with matrix solution (CHCA 9 mg·mL^−1^, 0.2% (*v*/*v*) TFA in 85% acetonitrile) containing two calibration standards, bradykinin 2–9 (30 pM·mL^−1^) and ACTH 18–39 (60 pM·mL^−1^), at a flow rate of 2.4 μL·min^−1^. A micro-fraction collector was used to deposit 1 mm spots every 2 s, and a total of 704 spots were collected in a 44 × 16 array for each nano LC run. The column was washed with a gradient (0–100–100% B for 5 min and 2 min, respectively, at a flow rate of 600 nL·min^−1^) and equilibrated to 0% B for 3.5 min before subsequent injections.

### 4.11. MALDI TOF/TOF Mass Spectrometry 

The fractionated samples were analyzed with a TOF/TOF 5800 System (SCIEX) instrument operated in the positive ion mode. The MALDI stage was set to continuous motion mode. MS data were acquired at 2800 laser intensity with 500 laser shots/spectrum (250 laser shots/sub-spectrum), and MS/MS data were acquired at 3700 laser intensity with a DynamicExit algorithm and a high spectral quality threshold or a maximum of 1000 laser shots/spectrum (250 laser shots/sub-spectrum). Up to 30 top precursors with S/N > 40 in the mass range 750–3500 Da were selected from each spot for MS/MS analysis.

### 4.12. Protein Identification

The Protein Pilot 5.0.1 software (SCIEX, Darmstadt, Germany) with the Paragon algorithm in thorough mode was used for the MS/MS spectra search against the *A. rubens* genome-derived protein database GCF_902459465.1 downloaded from the NCBI. Carbamidomethyl cysteine was set as a fixed modification. The database also incorporated a list of common contaminants. We accepted identification of the proteins having at least one peptide spectrum match (PSM) with the Protein Pilot peptide confidence ≥95%. Each protein should be identified at least in three replicates across four samples to be included in the final list of the accepted identification.

### 4.13. Bio-Layer Interferometry Assay

Samples of the frozen starfish tissues were thawed on ice, and approximately equal portions of each tissue from five different starfish individuals were mixed. Coelomocytes and coelomic epithelium were centrifuged at 200× *g* for 5 min at 4 °C and resuspended in lysis buffer (50 mM Hepes, pH 7.4, 150 mM NaCl, 40 mM DDM (Anatrace, D310), 0.2 mM CHS, 1 mM PMSF, and Sigmafast Protease Inhibitor Cocktail (Sigma, S8830)) at a rate of ~10 mg of tissue per 1 mL of lysis buffer. The body wall and arm tip was crushed in a cooled ceramic mortar filled by the lysis buffer. The samples were disintegrated by ultrasound at 150 watts for 4 min (cycles for 10 s impulses and 50 s rest) and incubated with stirring at 4 °C for 3 h. Cell lysate was clarified by centrifugation at 17,000× *g* for 40 min. Total protein concentration was measured using BCA assay. The clarified lysate was diluted to a concentration of 0.05 mg/mL in the lysis buffer and used as an analyte for BLI measurements on the Octet R2 instrument (Sartorius, Göttingen, Germany). Lyophilized 0.075 mg of recombinant Lystar5 sample was diluted in 200 µL of 10 mM acetate buffer, pH 4.0, and immobilized on the reactive 2nd generation biosensor (AR2G, ForteBio, 18-5092) using AR2G Reagent Kit (ForteBio, 18-5095) according to the manufacturer’s instructions. Empty biosensor blocked by ethanolamine was used for control measurements. Analyte samples of 200 µL were loaded to the 96-well plate, and binding (association) with the sensor was recorded during 900 s. Baseline (120 s) and dissociation (1800 s) were recorded in the same lysis buffer. Three control and experimental measurements were repeated for each of four tissues studied. Between measurements, the sensors were regenerated in the lysis buffer with an increase up to 1 M concentration of NaCl. BLI curve alignment was performed using Octet Analysis Studio 12.2 software (Sartorius). Data analysis was carried out using GraphPrism 8.0 (GraphPad Software).

### 4.14. Statistical Analysis 

Data are presented as mean ± SEM. Sample numbers (n) are indicated in the figure legends. Outliers were removed by ROUT test (Q = 1%). Before the comparisons, the data were tested for normality (Shapiro–Wilk test, at *p* = 0.05). The data were analyzed by the one-way ANOVA followed by Tukey’s or Dunnet’s test for normally distributed data and by the Kruskal–Wallis test followed by Dunn’s test for the data with non-Gaussian distribution as indicated in the figure legends. Differences in the data were considered statistically significant at *p* < 0.05. Analysis was performed using GraphPad Prism 8.0 software (GraphPad Software). The number of biological replicates was limited by availability of biological material. 

## 5. Conclusions

In conclusion, we analyzed the *Asterias rubens* genome and identified 48 TFPs, 39 of them with new sequences. Comparison of these proteins with human TFPs revealed the protein pairs with the high sequence homology, pointing to possible preservation of the function of some of them among different Metazoa phylums upon evolution. The high homology of Lystar5 proteins among echinoderms reflects its functional relevance. Integrin α-8-like protein was suggested as a target of Lystar5 in coelomic epithelium and the coelomocytes, although further study is required to confirm this hypothesis. Our study introduces the TFPs as a new and important class of regulatory proteins in starfish physiology.

## Data Availability

Proteomics dataset and supplementary FASTA files with protein sequences of human and *A. rubens* LU domains (LU_human.fasta and LU_arubens.fasta) are available on Zenodo https://doi.org/10.5281/zenodo.13983448 (accessed on 23 October 2024).

## Supplementary Materials

The following supporting information can be downloaded at: https://www.mdpi.com/article/10.3390/md22110488/s1, Figure S1: Heatmap of pairwise sequence similarities (%) calculated between all LU domains identified in the *Asterias rubens* starfish and LU domains of human Ly6/uPAR proteins; Figure S2: Three-finger protein sequences found in the *A. rubens* genome aligned with the most similar LU domain of human TFPs; Figure S3: Multiple sequence alignment of Lystar5 homologues from transcriptome databases of different Echinodermata species; Figure S4: Lystar5 mRNA level in different tissues of *Asterias amurensis* starfish; Figure S5: Gating strategy for flow cytometry experiments; Figure S6: BLI study of Lystar5 target in *A. amurensis* tissue lysates; Table S1: TFPs (Ly6/uPAR proteins) identified in the *A. rubens* genome; Table S2: Human Ly6/uPAR proteins from which LU domains were taken for comparison with *A. rubens* LU domains; Table S3: Similarity of Lystar5 homologues found in various Echinodermata species transcriptomes; Table S4: Primers were used of rtPCR for *A. rubens* TFPs mRNA analysis; Table S5: Proteins identified in elutions from Lystar5-conjugated or control NHS-Sepharose by proteomics.
