# Supplementary material for: In Search of the Role of Three-Finger Starfish Proteins
Source: Mar Drugs. 2024 Oct 30;22(11):488. doi: 10.3390/md22110488 (PMC11595613; doi:10.3390/md22110488)
Supplement: Supplementary file 1 [file marinedrugs-22-00488-s001.zip › marinedrugs-3257010-supplementary.pdf]

marine drugs

MDPI

Supplemantary

In Search of the Role of Three-Finger Starfish Proteins

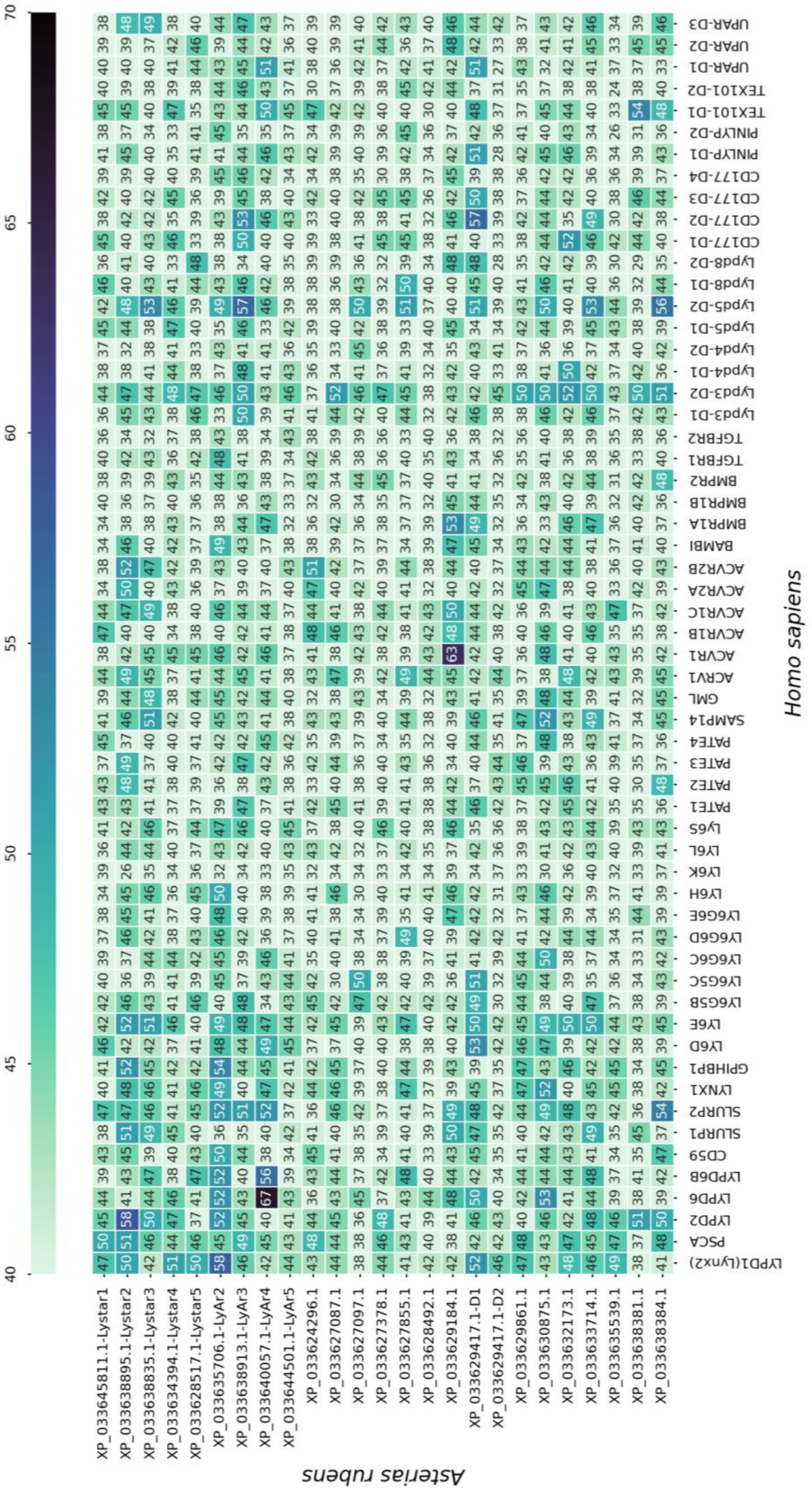

Figure S1. *Cont.*

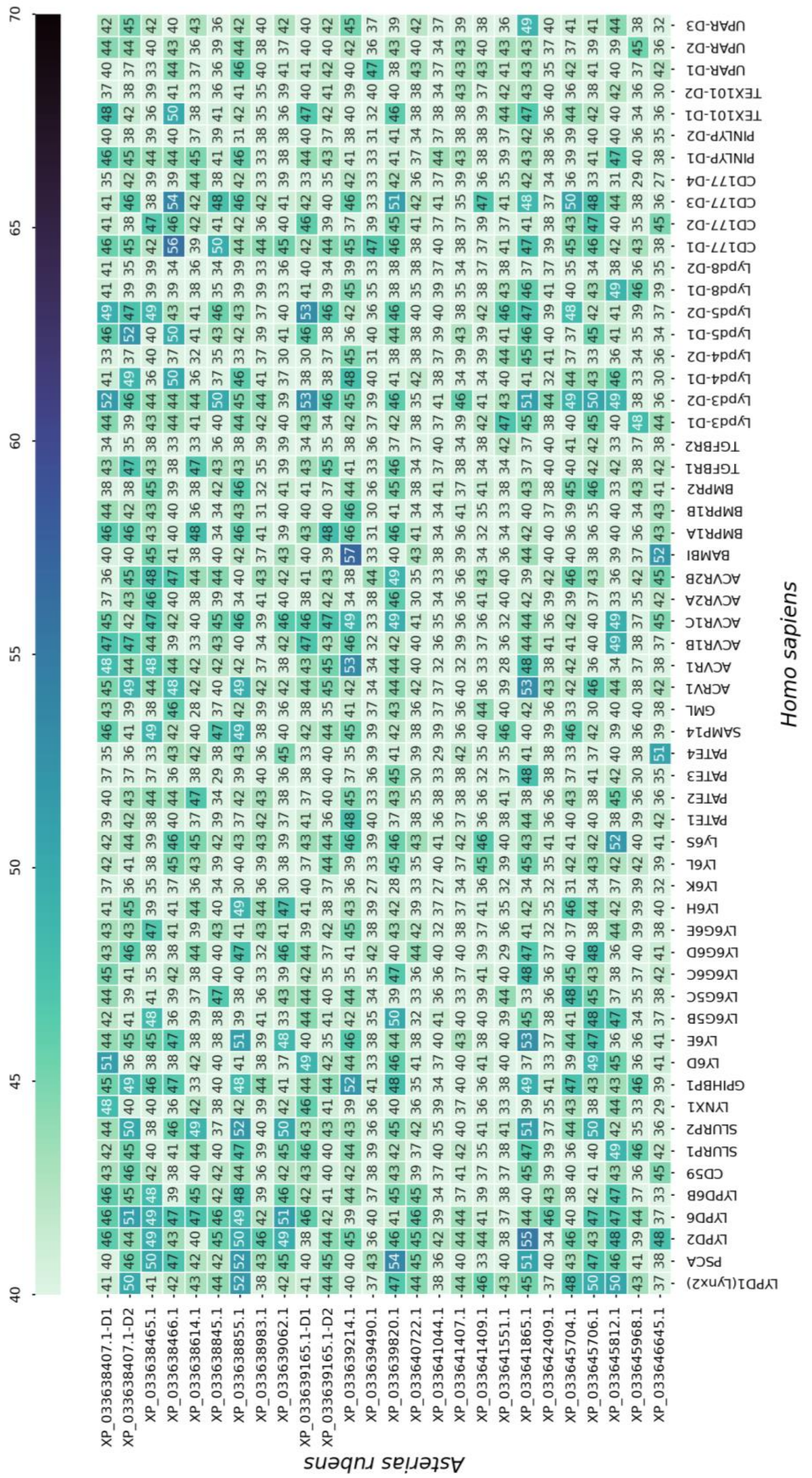

Figure S1. Heatmap of pairwise sequences similarities (%) calculated between all LU-domains identified in the *Asterias rubens* starfish and LU-domains of human Ly6/uPAR proteins. D1..D4 denotes different LU-domains for proteins with multiple LU-domains .

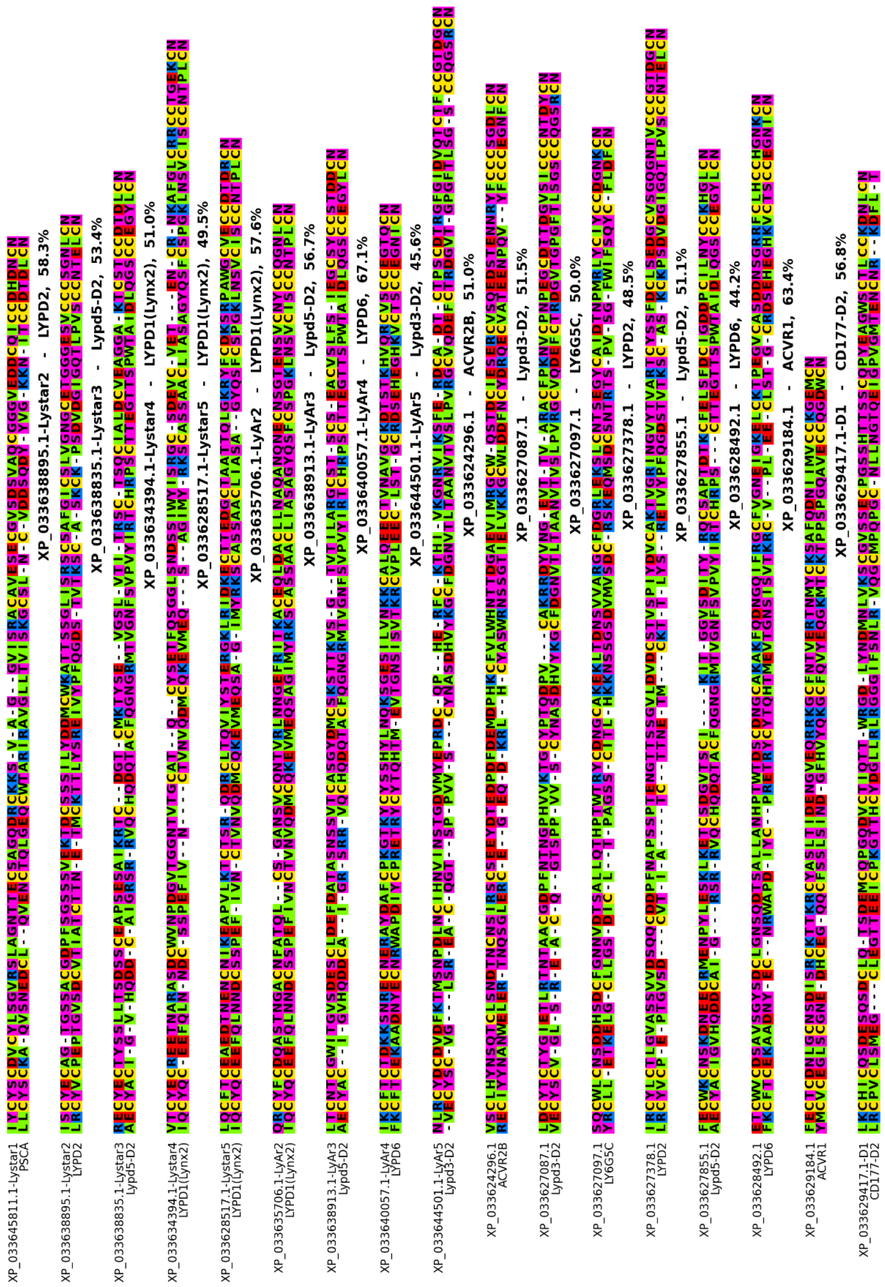

Figure S2. *Cont.*

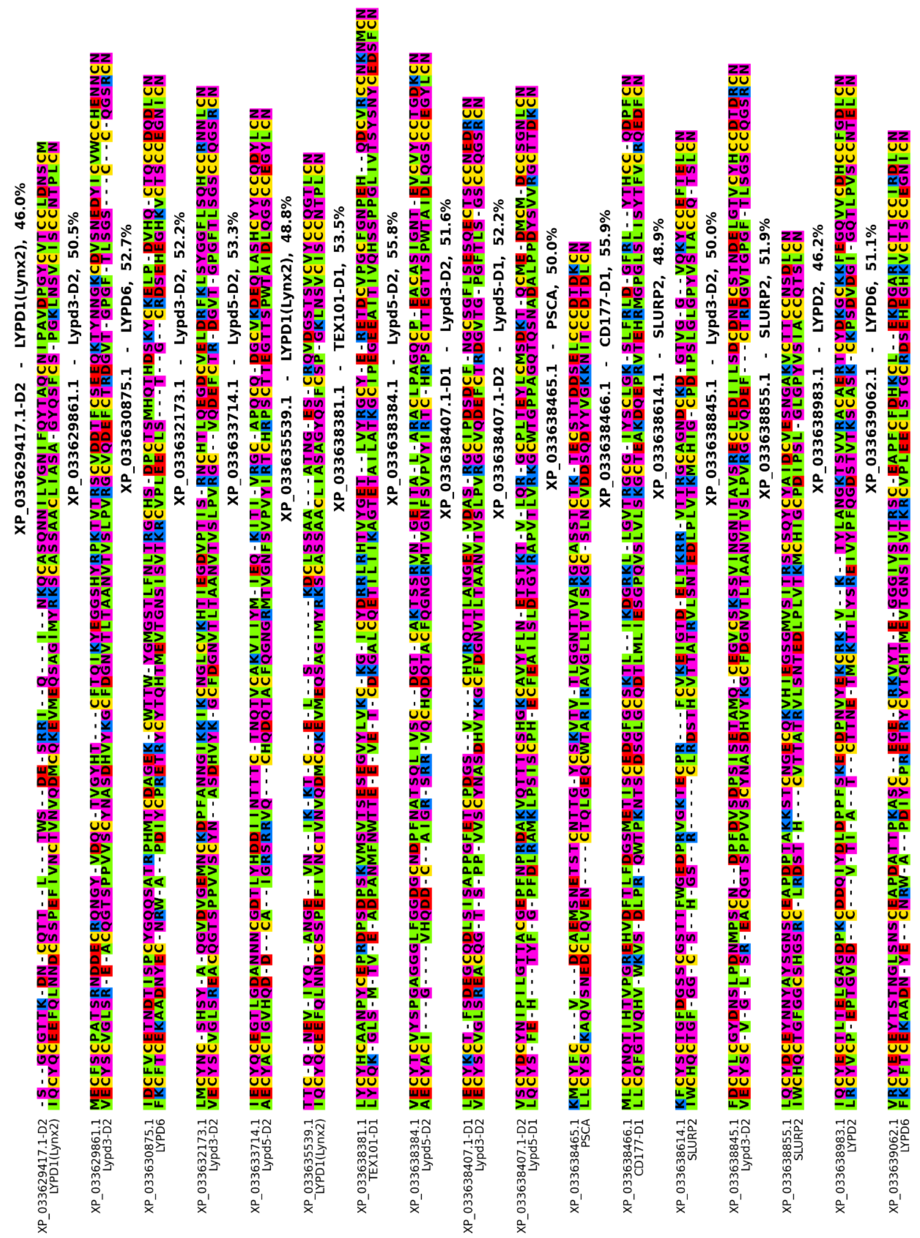

Figure S2. *Cont.*

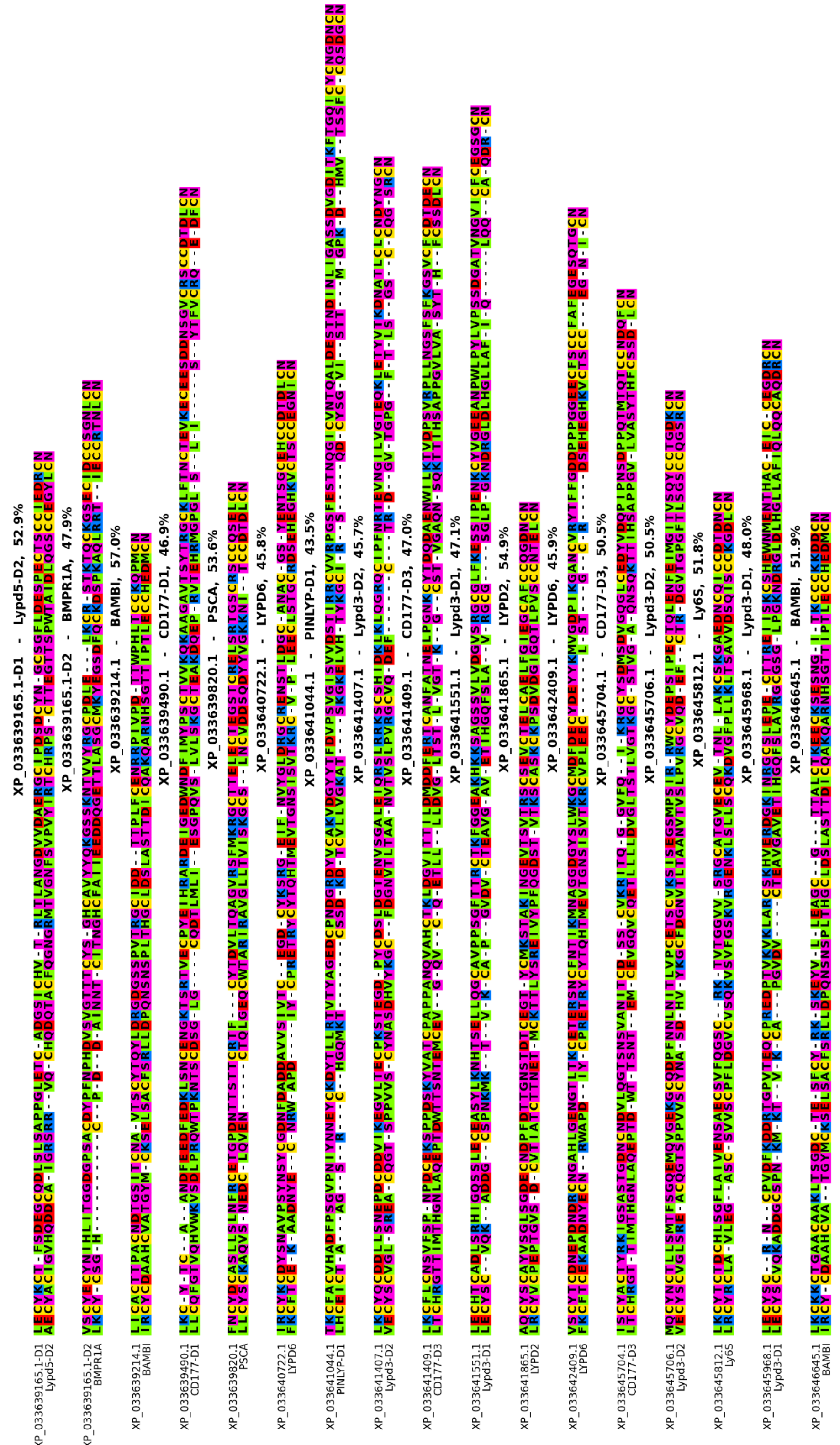

Figure S2. Three-finger proteins sequences found in the *A. rubens* genome aligned with most similar LU domain of human TFPs. The background of each residue symbol is colored depending on the type of side chain: hydrophobic (green), positively charged (blue), negatively charged (red), polar uncharged (magenta), cysteines - yellow. Human protein gene ID, accession number for the starfish protein, and sequence similarity (%) are shown on the panel. D1..D4 denotes different LU-domains for proteins with multiple LU-domains .

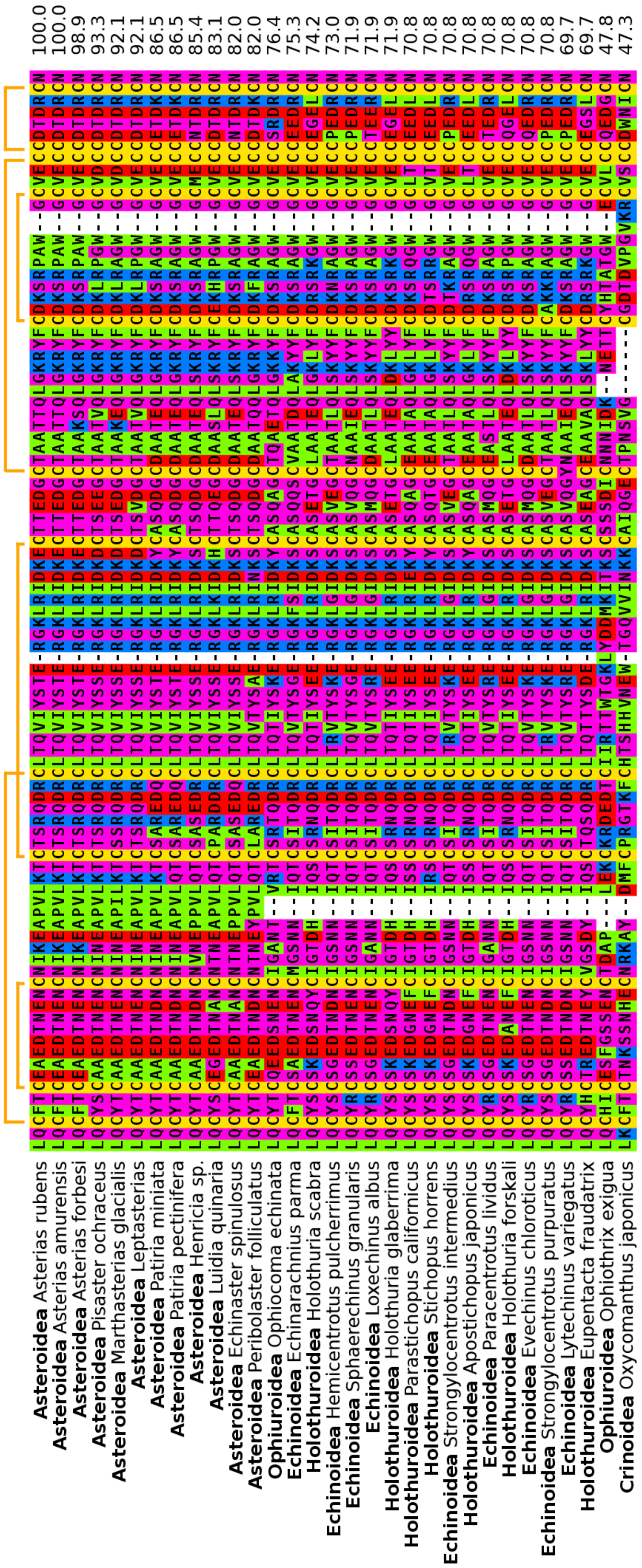

Figure S3. Multiple sequence alignment of Lystar5 homologues from transcriptomes databases of different Echinodermata species. The background of each residue symbol is colored depending on the type of side chain: hydrophobic (green), positively charged (blue), negatively charged (red), polar uncharged (magenta), cysteines - yellow.

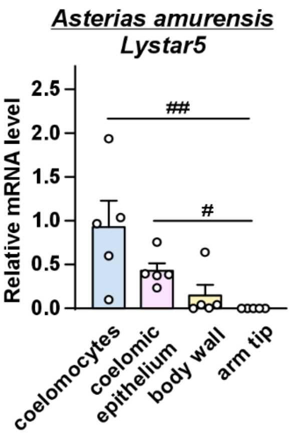

Figure S4. Lystar5 mRNA level in different tissues of *Asterias amurensis* starfish. Data presented as expression levels normalized to 40s ribosomal protein S13 and Glyceraldehyde-3-phosphate dehydrogenase mRNA ± SEM (n = 5). # ($p < 0.05$) and ## ($p < 0.01$) indicate difference between data groups according to Kruskal-Wallis test followed by Dunn's test.

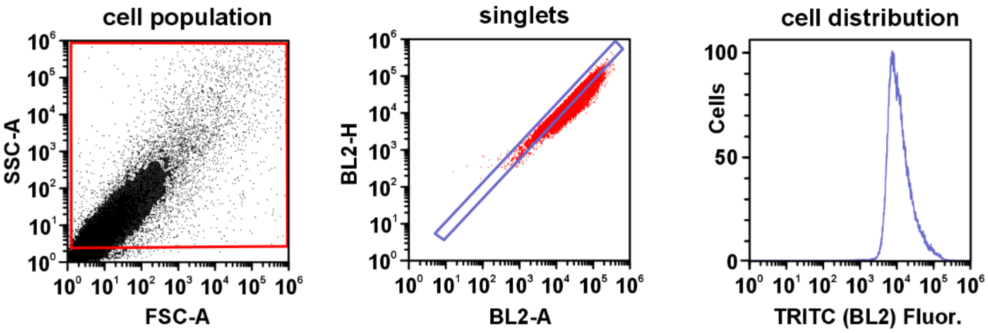

Figure S5. Gating strategy for flow cytometry experiments. The analysis of expression in coelomic epithelial cells is shown as an example.

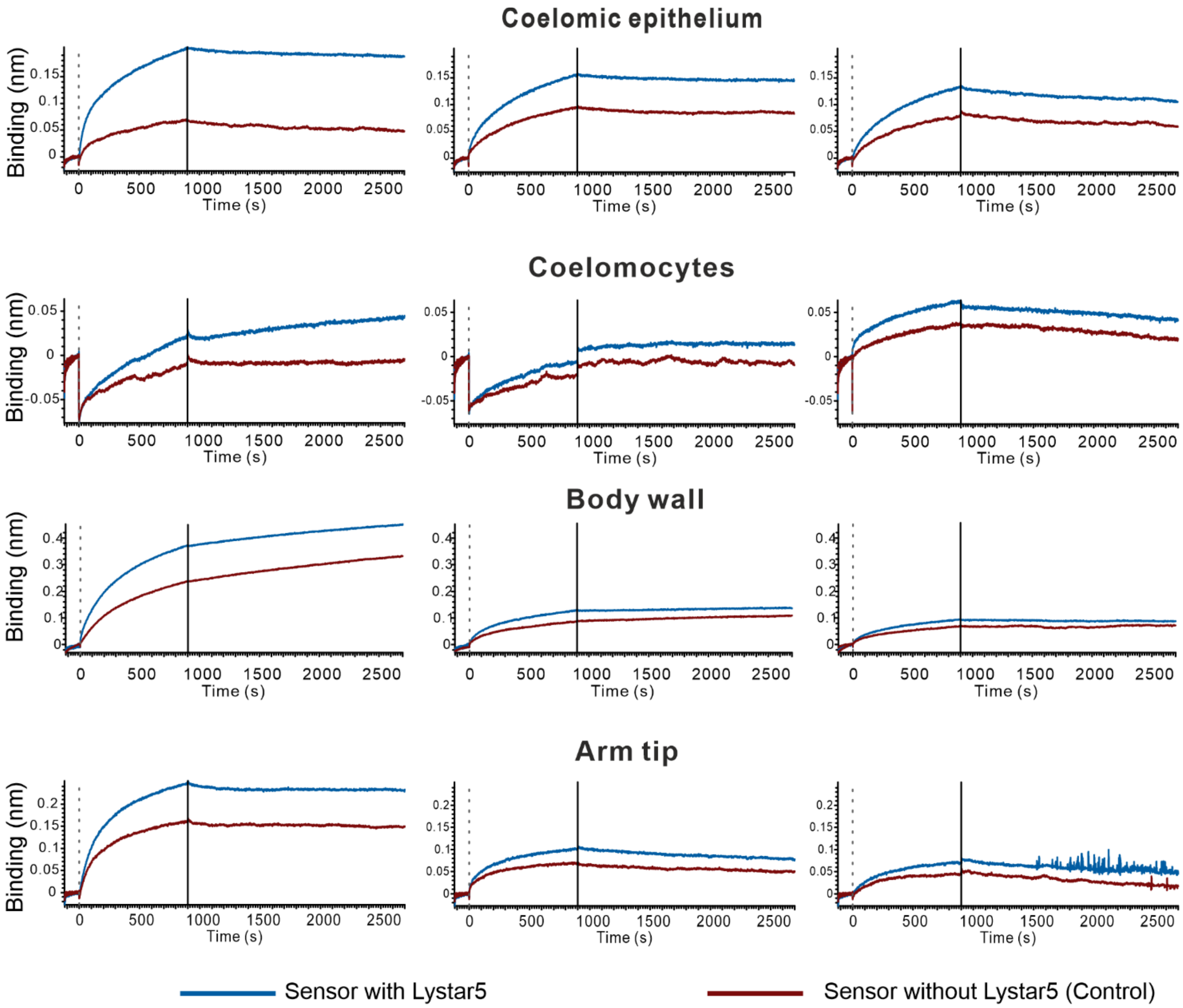

Figure S6. BLI study of Lystar5 target in *A. amurensis* tissues lysates. For each tissue (designed on panels) three repeats are shown. Each measurement consist baseline equilibration stage (Time < 0), association stage (from 0 to 900 seconds) and dissociation stage (>900 seconds). Sensograms from AR2G biosensor with immobilized recombinant Lystar5 (blue lines) and "empty" biosensor (brown line, without Lystar5 immobilization) for control unspecific binding are shown.

Table S1. TFPs (Ly6/uPAR proteins) identified in the *A. rubens* genome.

	Accession ID	Current description[1)]	Seq. length	Num. of LU domains	LU domain pos.	Signal[2)] peptide	TM[3)]	GPI[4)]
1	XP_033645811.1 Lystar1	uncharacterized protein LOC117305139	121	1	20-96	SP	0	GPI
2	XP_033638895.1 Lystar2	uncharacterized protein LOC117299466	126	1	20-102	SP	1	GPI
3	XP_033638835.1 Lystar3	ly-6/neurotoxin-like protein 1	124	1	22-100	SP	1	GPI
4	XP_033634394.1 Lystar4	uncharacterized protein LOC117295763	139	1	23-111	SP	1	GPI
5	XP_033628517.1-Lystar5	prostate stem cell antigen-like	151	1	34-123	SP	0	GPI
6	XP_033635706.1-LyAr2	adhesion G-protein coupled receptor G2-like	1113	1	224-305	SP	7	NO
7	XP_033638913.1-LyAr3	synergistic-like venom protein	126	1	20-103	SP	1	GPI
8	XP_033640057.1-LyAr4	ly6/PLAUR domain-containing protein 6-like	178	1	48-132	SP	0	GPI
9	XP_033644501.1-LyAr5	lymphocyte antigen 6D-like	152	1	27-120	SP	1	NO
10	XP_033624296.1	activin receptor type-2A-like	529	1	31-125	SP	1	NO
11	XP_033627087.1	uncharacterized protein LOC117289992	132	1	19-109	SP	0	GPI
12	XP_033627097.1	uncharacterized protein LOC117289999	138	1	23-114	SP	1	GPI
13	XP_033627378.1	uncharacterized protein LOC117290226	144	1	20-120	SP	0	GPI
14	XP_033627855.1	uncharacterized protein LOC117290533	127	1	21-104	SP	1	GPI
15	XP_033628492.1	uncharacterized protein LOC117291005	138	1	20-114	SP	1	GPI
16	XP_033629184.1	activin receptor type-1-like	536	1	37-107	NO	1	NO
17	XP_033629417.1	uncharacterized protein LOC117291678	232	2	24-108<br>134-200	SP	0	GPI
18	XP_033629861.1	uncharacterized protein LOC117291949	158	1	25-114	SP	1	NO
19	XP_033630875.1	mucin-5B-like	338	1	220-307	NO		GPI

	Accession ID	Current description[1)]	Seq. length	Num. of LU domains	LU domain pos.	Signal[2)] peptide	TM[3)]	GPI[4)]
20	XP_033632173.1	uncharacterized protein LOC117293828	141	1	27-114	SP	1	GPI
21	XP_033633714.1	uncharacterized protein LOC117295268	150	1	38-121	SP	0	NO
22	XP_033635539.1	adhesion G-protein coupled receptor G6-like	1022	1	186-244	SP	7	NO
23	XP_033638381.1	uncharacterized protein LOC117299074	139	1	25-115	SP	0	GPI
24	XP_033638384.1	uncharacterized protein LOC117299077	130	1	20-106	SP	0	GPI
25	XP_033638407.1	uncharacterized protein LOC117299091	240	2	21-102<br>111-195	SP	0	GPI
26	XP_033638465.1	uncharacterized protein LOC117299113	116	1	23-91	SP	0	GPI
27	XP_033638466.1	uncharacterized protein LOC117299114	132	1	24-107	SP	1	GPI
28	XP_033638614.1	uncharacterized protein LOC117299244	120	1	20-98	SP	0	GPI
29	XP_033638845.1	uncharacterized protein LOC117299416	139	1	24-114	SP	1	GPI
30	XP_033638855.1	ly-6/neurotoxin-like protein 1	122	1	20-98	SP	1	GPI
31	XP_033638983.1	uncharacterized protein LOC117299543	135	1	20-107	SP	1	GPI
32	XP_033639062.1	ly-6/neurotoxin-like protein 1	123	1	20-99	SP	1	GPI
33	XP_033639165.1	uncharacterized protein LOC117299718	266	2	21-102<br>111-199	SP	0	NO
34	XP_033639214.1	BMP and activin membrane-bound inhibitor homolog	231	1	30-104	SP	1	NO
35	XP_033639490.1	uncharacterized protein LOC117299968	173	1	40-146	SP	0	GPI
36	XP_033639820.1	uncharacterized protein LOC117300203	152	1	31-111	SP	0	GPI
37	XP_033640722.1	uncharacterized protein LOC117300963	130	1	21-108	SP	0	GPI
38	XP_033641044.1	uncharacterized protein LOC117301271	183	1	26-156	SP	1	GPI

	Accession ID	Current description[1)]	Seq. length	Num. of LU domains	LU domain pos.	Signal[2)] peptide	TM[3)]	GPI[4)]
39	XP_033641407.1	uncharacterized protein LOC117301495	157	1	19-133	SP	0	GPI
40	XP_033641409.1	uncharacterized protein LOC117301497	158	1	20-133	SP	1	GPI
41	XP_033641551.1	uncharacterized protein LOC117301641	181	1	37-157	SP	1	GPI
42	XP_033641865.1	uncharacterized protein LOC117302164	124	1	20-100	SP	0	GPI
43	XP_033642409.1	63 kDa sperm flagellar membrane protein-like	212	1	39-149	NO	0	GPI
44	XP_033645704.1	uncharacterized protein LOC117305083	147	1	29-124	SP	1	GPI
45	XP_033645706.1	uncharacterized protein LOC117305084	134	1	21-112	SP	0	GPI
46	XP_033645812.1	lymphocyte antigen 6E-like	121	1	20-96	SP	0	GPI
47	XP_033645968.1	uncharacterized protein LOC117305247	134	1	21-110	SP	1	GPI
48	XP_033646645.1	short neurotoxin 342-like	108	1	19-86	SP	0	GPI

[1)]According data in NCBI databases at Jun 2024.
[2)] Presence of signal peptide predicted by SignalP 6.0.
[3)] Transmembrane regions were identified by Phobius.
[4)] GPI anchoring was predicted by PredGPI.
Proteins which LU-domains were used for model building for HMMsearch (nums. 1-9) are selected by color.

Table S2. Human Ly6/uPAR proteins from which LU-domains were taken for comparison with *A. rubens* LU-domains (data given according to UNIPROT database at June 2024).

	Protein (gene) name	Uniprot accession	Description	Seq length	Num LU	LU domain position	GPI	Secreted form	TM
1	LYPD1 (Lynx2)	Q8N2G4	Ly6/PLAUR domain-containing protein 1	141	1	23-107	GPI		
2	PSCA	O43653	Prostate stem cell antigen	114	1	12-84	GPI		
3	LYPD2	Q6UXB3	Ly6/PLAUR domain-containing protein 2	125	1	23-100	GPI		
4	LYPD6	Q86Y78	Ly6/PLAUR domain-containing protein 6	171	1	47-128	GPI		
5	LYPD6B	Q8NI32	Ly6/PLAUR domain-containing protein 6B	183	1	60-141	GPI		
6	CD59	P13987	CD59 glycoprotein	128	1	26-95	GPI		
7	SLURP1	P55000	Secreted Ly-6/uPAR-related protein 1	103	1	23-100	NO	Sec	
8	SLURP2	P0DP57	Secreted Ly-6/uPAR domain-containing protein 2	97	1	23-95	NO	Sec	
9	LYNX1	P0DP58	Ly-6/neurotoxin-like protein 1	116	1	21-92	GPI	Sec	
10	GPIHBP1	Q8IV16	Glycosylphosphatidylinositol-anchored high density lipoprotein-binding protein 1	184	1	63-137	GPI		
11	LY6D	Q14210	Lymphocyte antigen 6D	128	1	21-93	GPI		
12	LY6E	Q16553	Lymphocyte antigen 6E	131	1	21-99	GPI		
13	LY6G5B	Q8NDX9	Lymphocyte antigen 6 complex locus protein G5b	201	1	26-105	NO	Sec	
14	LY6G5C	Q5SRR4	Lymphocyte antigen 6 complex locus protein G5c	150	1	63-140	NO	Sec	
15	LY6G6C	O95867	Lymphocyte antigen 6 complex locus protein G6c	125	1	20-98	GPI		
16	LY6G6D	O95868	Lymphocyte antigen 6 complex locus protein G6d	133	1	22-103	NO		1
17	LY6G6E	A0A0B4J1T7	Lymphocyte antigen 6 family member G6E	125	1	28-95	NO		1
18	LY6H	O94772	Lymphocyte antigen 6H	140	1	26-111	GPI		
19	LY6K	Q17RY6	Lymphocyte antigen 6K	165	1	52-122	GPI		
20	LY6L	H3BQJ8	Lymphocyte antigen 6L	138	1	28-109	GPI		
21	Ly6S	P0DTL4	Lymphocyte antigen 6S	134	1	27-105	GPI		
22	PATE1	Q8WXA2	Prostate and testis expressed protein 1	126	1	46-123	NO	Sec	
23	PATE2	Q6UY27	Prostate and testis expressed protein 2	113	1	29-108	NO	Sec	
24	PATE3	B3GLJ2	Prostate and testis expressed protein 3	98	1	21-95	NO	Sec	
25	PATE4	P0C8F1	Prostate and testis expressed protein 4	98	1	23-96	NO	Sec	
26	SAMP14	Q8TDM5	Sperm acrosome membrane-associated protein 4	124	1	21-97	GPI		
27	GML	Q99445	Glycosyl-phosphatidylinositol-anchored molecule-like protein	158	1	32-111	GPI		
28	ACRV1	P26436	Acrosomal protein SP-10	265	1	188-263	NO		1

	Protein (gene) name	Uniprot accession	Description	Seq length	Num LU	LU domain position	GPI	Secreted form	TM
29	ACVR1	Q04771	Activin receptor type-1	509	1	33-100	NO		1
30	ACVR1B	P36896	Activin receptor type-1B	505	1	32-102	NO		1
31	ACVR1C	Q8NER5	Activin receptor type-1C	493	1	26-93	NO		1
32	ACVR2A	P27037	Activin receptor type-2A	513	1	28-111	NO		1
33	ACVR2B	Q13705	Activin receptor type-2B	512	1	27-110	NO		1
34	BAMBI	Q13145	BMP and activin membrane-bound inhibitor homolog	260	1	28-105	NO		1
35	BMPR1A	P36894	Bone morphogenetic protein receptor type-1A	532	1	59-131	NO		1
36	BMPR1B	O00238	Bone morphogenetic protein receptor type-1B	502	1	30-103	NO		1
37	BMPR2	Q13873	Bone morphogenetic protein receptor type-2	1038	1	32-124	NO		1
38	TGFBR1	P36897	TGF-beta receptor type-1	503	1	34-107	NO		1
39	TGFBR2	P37173	TGF-beta receptor type-2	567	1	49-144	NO		1
40	Lypd3	O95274	Ly6/PLAUR domain-containing protein 3	346	2	31-114<br>138-222	GPI		
41	Lypd4	Q6UWN0	Ly6/PLAUR domain-containing protein 4	246	2	27-116<br>141-223	GPI		
42	Lypd5	Q6UWN5	Ly6/PLAUR domain-containing protein 5	251	2	26-109<br>133-214	GPI		
43	Lypd8	Q6UX82	Ly6/PLAUR domain-containing protein 8	237	2	20-107<br>123-201	GPI		
44	CD177	Q8N6Q3	CD177 antigen	437	4	22-111<br>131-210<br>209-300<br>323-400	GPI		
45	PINLYP	A6NC86	phospholipase A2 inhibitor and Ly6/PLAUR domain-containing protein	204	2	27-108<br>124-204	NO	Sec	
46	TEX101	Q9BY14	Testis-expressed protein 101	249	2	26-115<br>138-226	GPI		
47	UPAR	Q03405	Urokinase plasminogen activator surface receptor	335	3	23-99<br>115-199<br>214-294	GPI		

Table S3. Similarity of Lystar5 homologues found in various Echinodermata species transcriptomes.

Species	Class	Similarity with Lystar5 from A. rubens (%)	Accession ID
Asterias rubens	Asteroidea (starfishes)	100.0	GKCO01007097.1
Asterias amurensis		100.0	GAVL01016610.1
Asterias forbesi		98.9	GAUS01004065.1
Pisaster ochraceus		93.3	GAVN01030234.1
Marthasterias glacialis		92.1	GAVI01071235.1
Leptasterias sp.		92.1	GAVC01065371.1
Patiria miniata		86.5	HP097089.1
Patiria pectinifera		86.5	GFOQ01279557.1
Henricia sp.		85.4	GAVP01106615.1
Luidia quinaria		83.1	GJPY01010342.1
Echinaster spinulosus		82.0	GAVE01089069.1
Peribolaster folliculatus		82.0	GECB01035367.1
Ophiocoma echinata	Ophiuroidea (brittle stars)	76.4	GAUQ01041320.1
Ophiothrix exigua		47.8	GJOM01025453.1
Echinarachnius parma	Echinoidea (sea urchins)	75.3	GAVF01022245.1
Hemicentrotus pulcherrimus		73.0	IACU01036009.1
Sphaerechinus granularis		71.9	GAVR01034844.1
Loxechinus albus		71.9	GGVM01029998.1
Strongylocentrotus intermedius		70.8	GJVT01049422.1
Paracentrotus lividus		70.8	HACU01332369.1
Evechinus chloroticus		70.8	GAPB01045181.1
Strongylocentrotus purpuratus		70.8	GHFM01022636.1
Lytechinus variegatus		69.7	GAUR01071403.1
Holothuria scabra	Holothuroidea (sea cucumbers)	74.2	GIRH01044451.1
Holothuria glaberrima		71.9	GIVL01335762.1
Parastichopus californicus		70.8	GAVO01024495.1
Stichopus horrens		70.8	HAMZ01053418.1

Species	Class	Similarity with Lystar5 from A. rubens (%)	Accession ID
Apostichopus japonicus		70.8	HADF01084390.1
Holothuria forskali		70.8	GIPR01005287.1
Eupentacta fraudatrix		69.7	GHCL02044331.1
Oxycomanthus japonicus	Crinoidea (sea lilies)	47.3	GAZO01033255.1

Table S4. Primers were used of rtPCR for *A. rubens* TFPs mRNA analysis.

Protein (mRNA ID)	Forward primer	Reverse primer	Melting tempera-ture, °C	Am-plicon length, b.p.
40s ribosomal pro-tein S13 (XM_033789754.1)	TCTCCTCTTCGGCCTTACCT	TGGGAGTCACGCAAGATCAC	84.4	154
Glyceraldehyde-3-phosphate dehydrogenase (XM_033786142.1)	GCTGATGCCCCAATGTTTGTT	TAATGGTGCAAGGCAGTTC	80	136
Lystar5 (XM_033772626.1)	TTGTAAAAGGTTG	GGCAATAAAAAG	91	584
LyAr2 (XM_033779815.1)	ACCGCGTAGCTTGACTTGTG	GGCTGACAAACTGAGTCCCA	85.3	123
LyAr3 (XM_033783022.1)	TGAGTGCCCTGACGTG-TAAC	AGAGGTTGAGCAACCACGAG	85.5	188
LyAr4 (XM_033784166.1)	CCGTTCGTCGTTGCGATAGT	CCTCGGCCCAGAGAAACAAG	84	206

LyAr5 (XM_033788610.1)	ATTGCCAACCACATGAACGC	CCATCTGTACCGCAGCAGAA	85.9	163
Integrin α-8-like protein (XM_033788610.1)	ACACAGTGGCTTTCCACAGG	CCGATACAGGGCAACGGTAG	86.4	120

Table S5. Proteins identified in elutions from Lystar5-conjugated or control NHS-Sepharose by proteomics (MS/MS counts). TM – transmembrane segments, SP – signal peptide.

N	Accession	Protein annotation (according database)	Seq. length	TM	SP	# of peptides identified							
						control resin				Lystar5 resin			
						1	2	3	4	1	2	3	4
**Proteins identified only in Lystar5 resin but not in the control**													
**1**	XP_033647246.1	Transketolase-like	630	–	–	0	0	0	0	66	50	68	0
**2**	XP_033633626.1	Arginine kinase-like	440	–	–	0	0	0	0	62	8	28	0
**3**	XP_033646271.1	Glycogen phosphorylase, brain form-like	862	–	–	0	0	0	0	51	9	22	0
**4**	XP_033640157.1	Vasodilator-stimulated phosphoprotein-like	538	–	–	0	0	0	0	47	19	21	34
**5**	XP_033644648.1	Digestive cysteine proteinase 1-like	553	–	+	0	0	0	0	38	16	53	14
**6**	XP_033638450.1	Vinculin-like	1258	–	–	0	0	0	0	20	19	68	0
**7**	XP_033635206.1	SH3 domain-binding glutamic acid-rich-like protein 3	92	–	–	0	0	0	0	13	12	35	0
**8**	XP_033639394.1	Integrin alpha-8-like	1051	+	+	0	0	0	0	13	23	11	0
**9**	XP_033629014.1	Actin-related protein 3	418	–	–	0	0	0	0	11	19	13	0
**10**	XP_033630706.1	WW domain-binding protein 2-like	261	–	–	0	0	0	0	11	12	14	0
**11**	XP_033635425.1	Homogentisate 1,2-dioxygenase-like	438	–	–	0	0	0	0	10	9	24	0
**Proteins identified both in control and Lystar5 resins**													
**12**	XP_033638835.1	Ly-6/neurotoxin-like protein 1	171	+	–	529	359	594	459	320	507	978	348
**13**	XP_033640031.1	Actin, cytoplasmic	376	–	–	124	139	173	135	1195	312	435	135

N	Accession	Protein annotation (according database)	Seq. length	TM	SP	# of peptides identified							
						control resin				Lystar5 resin			
						1	2	3	4	1	2	3	4
14	XP_033625559.1	Uncharacterized protein LOC117288794	645	–	–	119	63	152	121	102	151	132	130
15	XP_033644716.1	Melanotransferrin-like	751	+	+	103	49	138	116	230	333	455	97
16	XP_033637954.1	Histone H3, embryonic-like	249	–	–	76	115	92	49	83	121	71	77
17	XP_033640043.1	Neo-calmodulin-like	140	–	–	53	0	0	41	18	16	24	30
18	XP_033630725.1	Histone H2B-like	124	–	–	49	50	53	35	61	66	71	22
19	XP_033647699.1	Cytosolic 10-formyltetrahydrofolate dehydrogenase-like	923	–	–	48	33	34	52	316	125	217	65
20	XP_033647973.1	Ras-like GTP-binding protein RHO	320	–	–	47	21	43	40	48	21	37	22
21	XP_033641517.1	Profilin-like	139	–	–	42	0	26	39	210	30	44	8
22	XP_033646748.1	Collagen alpha-5(VI) chain-like	568	+	+	39	87	73	50	135	78	62	58
23	XP_033639733.1	ATP synthase subunit beta, mitochondrial	526	–	+	36	56	49	45	68	37	81	53
24	XP_033634546.1	ATP synthase subunit alpha, mitochondrial-like	555	–	+	26	0	12	22	24	38	58	0
25	XP_033646766.1	Guanine nucleotide-binding protein G(i) subunit alpha	354	–	–	26	0	31	16	26	28	37	14
26	XP_033647009.1	Ras-related protein Rab-7a	205	–	+	21	0	11	19	24	27	40	8
27	XP_033639529.1	Integrin alpha-9-like	1097	+	+	19	13	0	0	49	55	57	0
28	XP_033625392.1	Ras-related protein Rab-8A-like	208	–	–	17	0	10	0	42	15	31	0
29	XP_033633870.1	Tropomyosin-like	284	–	–	17	7	21	9	55	42	41	13
30	XP_033643108.1	Histone H2A-like	138	–	–	15	57	37	39	29	68	47	10
31	XP_033643955.1	Glutamate dehydrogenase, mitochondrial-like	536	–	–	15	0	0	0	107	64	100	0
32	XP_033631175.1	Actin-related protein 2/3 complex subunit 1A-like	372	–	–	12	0	0	27	48	0	15	29

N	Accession	Protein annotation (according database)	Seq. length	TM	SP	# of peptides identified							
						control resin				Lystar5 resin			
						1	2	3	4	1	2	3	4
33	XP_033634922.1	Dual oxidase 1-like	1660	7	+	11	0	0	0	35	45	72	0
34	XP_033642033.1	Glyceraldehyde-3-phosphate dehydrogenase-like	334	–	–	11	0	14	16	78	28	44	0
35	XP_033643903.1	Gelsolin-like protein 2	364	–	–	11	34	31	12	150	26	58	12
36	XP_033635343.1	ATP synthase subunit O, mitochondrial-like	209	–	–	10	0	0	0	8	8	9	0
37	XP_033639284.1	Integrin beta-1-like	830	+	+	10	6	0	0	10	46	12	0
38	XP_033641837.1	6-phosphogluconate dehydrogenase, decarboxylating-like	482	–	–	10	0	0	0	69	7	11	0
39	XP_033627076.1	Fructose-bisphosphate aldolase, muscle type-like	363	–	–	9	25	17	0	80	0	28	0
40	XP_033640449.1	Cathepsin B-like	327	–	+	9	0	14	8	37	24	7	0
41	XP_033641324.1	Uncharacterized protein LOC117301444	301	–	+	9	10	11	0	0	20	25	0
42	XP_033647181.1	Actin-related protein 2/3 complex subunit 4	168	–	–	8	9	9	8	25	9	8	7
43	XP_033642716.1	Endoplasmic reticulum chaperone bip-like	666	–	+	0	25	0	0	51	9	0	7
44	XP_033638384.1	Uncharacterized protein LOC117299077	130	–	+	0	20	15	0	47	22	47	0
45	XP_033638930.1	Tubulin beta-4B chain-like	446	–	–	0	14	0	0	26	0	19	19
46	XP_033629661.1	Advillin-like	839	–	–	0	11	10	0	131	7	18	10
**47**	**XP_033628517.1**	**Lystar5 (prostate stem cell antigen-like)**	**155**	**–**	**+**	**0**	**7**	**12**	**12**	**899**	**1023**	**2001**	**778**
48	XP_033630534.1	Fascin-like	508	–	–	0	0	15	8	175	32	96	0
49	XP_033643951.1	Filamin-A-like	2789	+	–	0	0	10	0	97	73	50	10
50	XP_033644507.1	Gelsolin-like protein 2	362	–	–	0	0	10	7	104	16	32	7
51	XP_033647349.1	Ras-related protein Rap-1b-like	191	–	–	0	0	9	0	10	23	47	0

N	Accession	Protein annotation (according database)	Seq. length	TM	SP	# of peptides identified							
						control resin				Lystar5 resin			
						1	2	3	4	1	2	3	4
**52**	XP_033647910.1	ATP synthase subunit gamma, mitochondrial-like	272	–	–	0	0	0	10	10	0	21	8
**53**	XP_033627690.1	Major vault protein-like	859	–	–	0	0	0	7	76	24	75	0

Disclaimer/Publisher's Note: The statements, opinions and data contained in all publications are solely those of the individual author(s) and contributor(s) and not of MDPI and/or the editor(s). MDPI and/or the editor(s) disclaim responsibility for any injury to people or property resulting from any ideas, methods, instructions or products referred to in the content.
